# Supplementary material for: Accurate prediction of personalized olfactory perception from large-scale chemoinformatic features
Source: Gigascience. 2017 Dec 15;7(2):1–11. doi: 10.1093/gigascience/gix127 (PMC5824779; doi:10.1093/gigascience/gix127)
Supplement: GIGA-D-17-00082_Original_Submission.pdf [file gix127_giga-d-17-00082_original_submission.pdf]

GigaScience

Accurate Prediction of Personalized Olfactory Perception from Large-Scale Chemoinformatic Features

--Manuscript Draft--

Manuscript Number:	GIGA-D-17-00082
Full Title:	Accurate Prediction of Personalized Olfactory Perception from Large-Scale Chemoinformatic Features
Article Type:	Research
Funding Information:	National Science Foundation (1452656) — Dr. Yuanfang Guan<br>Alzheimer's Association (BAND-15-367116) — Dr. Yuanfang Guan
Abstract:	Background<br>The olfactory stimulus-percept problem has been studied for more than a century, yet it is still hard to precisely predict the odor given the large-scale chemoinformatic features of an odorant molecule. A major challenge is that the perceived qualities vary greatly among individuals due to different genetic and cultural backgrounds. Moreover, the combinatorial interactions between multiple odorant receptors and diverse molecules significantly complicate the olfaction prediction. Many attempts have been made to establish structure-odor relationships for intensity and pleasantness, but no models are available to predict the personalized multi-odor attributes of molecules. In this study, we describe our winning algorithm for predicting individual and population perceptual responses to various odorants in DREAM Olfaction Prediction Challenge.<br>Results<br>We find that random forest model consisting of multiple decision trees is well-suited to this prediction problem, given the large feature spaces and high variability of perceptual ratings among individuals. Integrating both population and individual perceptions into our model effectively reduces the influence of noise and outliers. By analyzing the importance of each chemical feature, we find that a small set of low- and non-degenerative features is sufficient for accurate prediction.<br>Conclusions<br>Our random forest model successfully predicts personalized odor attributes of structurally diverse molecules. This model together with the top discriminative features has the potential to extend our understanding of olfactory perception mechanisms and provide an alternative for rational odorant design.
Corresponding Author:	Yuanfang Guan<br><br>UNITED STATES
Corresponding Author Secondary Information:	
Corresponding Author's Institution:	
Corresponding Author's Secondary Institution:	
First Author:	Hongyang Li
First Author Secondary Information:	
Order of Authors:	Hongyang Li
	Bharat Panwar
	Gilbert S. Omenn
	Yuanfang Guan
Order of Authors Secondary Information:	
Opposed Reviewers:	

Powered by Editorial Manager® and ProduXion Manager® from Aries Systems Corporation

Additional Information:	
**Question**	**Response**
Are you submitting this manuscript to a special series or article collection?	No
**Experimental design and statistics**<br><br>Full details of the experimental design and statistical methods used should be given in the Methods section, as detailed in our Minimum Standards Reporting Checklist. Information essential to interpreting the data presented should be made available in the figure legends.<br><br>Have you included all the information requested in your manuscript?	Yes
**Resources**<br><br>A description of all resources used, including antibodies, cell lines, animals and software tools, with enough information to allow them to be uniquely identified, should be included in the Methods section. Authors are strongly encouraged to cite Research Resource Identifiers (RRIDs) for antibodies, model organisms and tools, where possible.<br><br>Have you included the information requested as detailed in our Minimum Standards Reporting Checklist?	Yes
**Availability of data and materials**<br><br>All datasets and code on which the conclusions of the paper rely must be either included in your submission or deposited in publicly available repositories (where available and ethically appropriate), referencing such data using a unique identifier in the references and in the “Availability of Data and Materials” section of your manuscript.<br><br>Have you have met the above requirement as detailed in our Minimum Standards Reporting Checklist?	Yes

Powered by Editorial Manager® and ProduXion Manager® from Aries Systems Corporation

Manuscript

Click here to download Manuscript manuscript.docx

Accurate Prediction of Personalized Olfactory Perception from Large-Scale Chemoinformatic Features

Hongyang Li [1], Bharat Panwar [1], Gilbert S. Omenn [1,2], Yuanfang Guan [1,*]

1. Department of Computational Medicine and Bioinformatics, University of Michigan, 100 Washtenaw Avenue, Ann Arbor, MI 48109, USA

2. Departments of Internal Medicine and Human Genetics and School of Public Health, University of Michigan, Ann Arbor, MI 48109, USA

* Corresponding author: gyuanfan@umich.edu

Keywords: Olfactory Perception, Structure-Odor Relationships, Random Forest, Chemoinformatics

Abstract

Background

The olfactory stimulus-percept problem has been studied for more than a century, yet it is still hard to precisely predict the odor given the large-scale chemoinformatic features of an odorant molecule. A major challenge is that the perceived qualities vary greatly among individuals due to different genetic and cultural backgrounds. Moreover, the combinatorial interactions between multiple odorant receptors and diverse molecules significantly complicate the olfaction prediction. Many attempts have been made to establish structure-odor relationships for intensity and pleasantness, but no models are available to predict the personalized multi-odor attributes of molecules. In this study, we describe our winning algorithm for predicting individual and population perceptual responses to various odorants in DREAM Olfaction Prediction Challenge.

Results

We find that random forest model consisting of multiple decision trees is well-suited to this prediction problem, given the large feature spaces and high variability of perceptual ratings among individuals. Integrating both population and individual perceptions into our model effectively reduces the influence of noise and outliers. By analyzing the importance of each chemical feature, we find that a small set of low- and non-degenerative features is sufficient for accurate prediction.

Conclusions

Our random forest model successfully predicts personalized odor attributes of structurally diverse molecules. This model together with the top discriminative features has the potential to extend our understanding of olfactory perception mechanisms and provide an alternative for rational odorant design.

Background

Olfactory perception is the sense of smell in the presence of odorants. The odorants bind to and activate olfactory receptors (ORs), which transmit the signal of odor to the brain [1]. The existence of a large family of olfactory receptors enables humans to perceive an enormous variety of odorants with distinct sensory attributes [1]. An olfactory receptor can respond to multiple odor molecules; conversely, an odorant may interact with many olfactory receptors with different affinities [2]. Unlike the well-defined wavelength of light in vision and frequency of sound in hearing, the size and dimensionality of the olfactory perceptual space is still unknown [3]. It is not clear how the numerous physicochemical properties of a molecule relate to its odor, and how mammals process and detect the broad range of the olfactory spectrum. Some structurally similar compounds display distinct odor profiles, whereas some dissimilar molecules exhibit almost the same smell [4–6]. Even for an identical molecule, the perceived quality varies immensely between individuals due to genetic variation [7]. Therefore, accurate prediction of personalized olfactory perception from the chemical features of a molecule is highly challenging.

In the past, many attempts have been made to establish structure-odor relationships and predict the odor from the physicochemical properties of a molecule [8]. An early study showed that volatile and lipophilic molecules fulfill the requirements to be odorants [9]. The correlation of odor intensities with different structural, topological and electronic descriptors was calculated for 58 different odorants; molecular weight, partial charge on most negative atom, quantum chemical polarity parameter, average distance sum connectivity and a measure of the degree of unsaturation were particularly important descriptors [10]. Multi-dimensional scaling and self-organizing maps were used to produce two-dimensional maps of the Euclidean approximation of olfactory perception space [11]. A principal component analysis identified the latent variables in a semantic odor profile database of 881 perfume materials with semantic profiles of 82 odor

descriptors and classified odors into 17 different classes [12]. Although it is not possible to predict the odor profile of a molecule, some progress has been achieved for predicting the intensity [13] and pleasantness of an odorant. Methods for predicting perceived pleasantness of an odorant have utilized the most correlated physical features of molecular complexity [14] and molecular size [15,16]. A major challenge is that different individuals perceive odorants with different sets of odorant receptors [17,18], and perception is also strongly shaped by learning and experience [19]. Different cultures have different linguistic descriptions of smells, so generating olfaction datasets is tedious work. Many computational methods have been developed to relate chemical structure to percept [4,10,15,16,20–23] but most of them are based on single and very old psychophysical datasets [24]. Therefore, a rigorous quantitative structure-activity relationship (QSAR) model [25,26] of personalized olfactory perception is needed for accurate predictions.

The Dialogue on Reverse Engineering Assessment and Methods (DREAM) organized the olfaction prediction challenge [27]. DREAM is a leader in organizing crowdsourcing challenges to evaluate model predictions and algorithms in systems biology and medicine [28]. Here we describe our winning algorithm, the best-performer of sub-challenge 1 for predicting individual responses and the second best-performer of sub-challenge 2 for predicting population responses. Since olfactory perception is inherently a complex non-linear process, decision tree based algorithms are well suited to this problem. Particularly, random forest (RF) consisting of multiple decision trees addresses the overfitting issue, when the feature space is much larger than the sample space. Moreover, random forest is relatively robust to noise and outliers [29], especially when large variability of individual perceptual responses is observed. To further reduce the effects of large variability, noise and outliers, we integrated the average rating of individuals (population response) into our model. Our final model succeeds in predicting olfactory perception using only a small set of chemical features. These features are likely to be

low- and non-degenerative molecular descriptors, indicating that traditional simple descriptors like functional groups are less effective in distinguishing the odor profiles of structurally similar molecules. Meanwhile, our model potentially provides useful insights on the basic molecular mechanisms of olfactory perception. Together with new scaffolds of odorants observed in the dataset and top discriminative chemoinformatic features, our model offers an alternative for rational odorant design.

Data Description

Psychophysical dataset

The DREAM organizers provided psychophysical data that were originally collected between February 2013 and July 2014 as part of the Rockefeller University Smell Study [30]. The data was collected from 61 ethnically diverse healthy men and women between the ages of 18 and 50. These subjects were naïve and didn't receive any kind of olfaction training. In the DREAM olfaction prediction challenge, the data of only 49 subjects was provided because some subjects didn't give permission to use their data. The perceptual ratings of 476 different molecules were assigned by these 49 subjects at two different concentrations (high and low); in addition, 20 molecules were tested twice. Each subject rated the perception of 992 stimuli (476 plus 20 replicated molecules at two different concentrations). Twenty-one perceptual attributes (intensity, pleasantness, and 19 semantic attributes) were used to describe the odor profile of a molecule. The semantic attributes are: bakery, sweet, fruit, fish, garlic, spices, cold, sour, burnt, acid, warm, musky, sweaty, ammonia/urinous, decayed, wood, grass, flower, and chemical. Subjects used a scale from 0 to 100 where 0 is "extremely weak" and 100 is "extremely strong" for intensity; 0 is "extremely unpleasant" and 100 is "extremely pleasant" for pleasantness; 0 is "not at all" and 100 is "very much" for semantic attributes. This dataset of 476 chemicals was divided into three subsets by the organizers: 338 for the training set, 69 for the leaderboard, and 69 for the test set. We combined the 338 training and 69 leaderboard molecules (407 molecules in total) as our final training set.

Chemoinformatic features of molecules

The participating investigators were encouraged to use any kind of chemical and physical properties of the molecules for developing prediction models. By default, the organizers

provided 4,884 different chemical features for each of the 476 molecules, calculated by a commercial chemoinformatics software package known as Dragon (version 6) [31]. Features were divided into 29 different logical molecular descriptors blocks including Constitutional descriptors, Topological indices, 2D autocorrelations, etc. These chemoinformatic features are useful in establishing structure-odor relationships and further developing machine learning prediction models. The compound identification number (CID) for each molecule was also provided so participating investigators could obtain more information about the molecules from other resources (e.g., PubChem).

Results

The overall workflow of the olfaction prediction is shown in **Figure 1**. The organizers provided an unpublished large psychophysical dataset of 476 structurally and perceptually diverse molecules sensed by 49 different individuals [30]. Twenty-one perceptual attributes were collected, including odor intensity, pleasantness and 19 semantic descriptors. A subset of 407 molecules (338 training and 69 leaderboard molecules) was used as the final training set in our random forest model and the other 69 held-out molecules formed the test set. The organizers provided the Dragon software [31] based large-scale molecular descriptors, containing 4,884 chemical features for each molecule. Models were evaluated based on the Pearson's correlation between the observed and predicted perceptions.

Variability of olfactory perception among individuals

The intensity perceptions of 476 molecules at high and low concentrations vary tremendously among individuals. For example, individuals 10, 29 and 46 exhibit entirely different perceptual profiles for intensity (**Figure 2**). Ideally the perceptual rating for intensity should increase as the measuring concentration rises (blue lines in **Figure 2A**), while it is commonly observed that the intensity rating of some molecules decreases (red lines in **Figure 2A** and **Supplementary Figure 1**). In fact, the 49 subjects were lacking any kind of professional training and they were biased in assigning the perceptual rating value between 0 and 100 (**Figure 2B** and **Supplementary Figure 2**). Except for molecules without odor rated near 0, individual 10 tended to assign rating uniformly, whereas individual 29 preferred to rate at 100 and individual 46 was inclined to rate around 50.

In addition to intensity, the other perceived attributes were rated differently among individuals (**Figure 2C**). Even for the same molecule, cyclopentanethiol (CID: 15510), sixteen subjects did not apply the descriptor “garlic”, whereas nine subjects rated it 100. Similarly, 2-acetylpyridine (CID: 14286) showed great variability in “warm” ratings - about half of the subjects rated it 0 and the other half perceived “warm” from it. The large differences may result from the relative ambiguity of the word “warm” to describe odor. The average variance of 21 attribute ratings across all individuals is shown in **Figure 2D**. Compared to intensity and pleasantness, the 19 semantic qualities display much larger coefficients of variation. Thus, the diversity of perceptual ratings between subjects considerably complicates the prediction challenge.

Strategies for accurate personalized olfaction predictions

Considering the large variability of the perceived ratings, we propose that random forest could be an excellent choice as a base learner, because it applies the strategy of training on different parts of the dataset and averaging multiple decision trees to reduce the variance and avoid overfitting. We compared different machine learning algorithms (linear, ridge, support vector regression, random forest) using 5-fold cross validations, and found that random forest outperforms other base learners in predicting individual responses for “intensity”, “pleasantness” and 19 semantic descriptors (**Figure 3A**). Given a small sample size of 407 training molecules, random forest identifies and utilizes the most discriminative features out of 4,884 molecular descriptors to make decisions. Clearly, a simple linear regression model fails when the dimension of the feature space is too large. Therefore, random forest was selected as our base-learner and used in the follow-up improvements.

Recognizing the population responses (the average perceptions of all individuals) are more stable compared with individual responses, we overcome the variability of individual ratings by

introducing a weighting factor α. This parameter serves as a balance between individual and population ratings. When α equals 0, only population ratings are considered. Conversely, when α equals 1, only individual ratings are used (See **Methods**). Surprisingly, a small α = 0.2 achieves the largest Pearson's correlation coefficient (**Figure 3B**). Without population information (α = 1.0), the correlation of predicting the 19 semantic descriptors is the lowest. This reveals that population perceptions play a crucial role when individual responses display large fluctuations.

To further improve the performance, we applied a sliding window of 4-letter size to each molecule name, generating a total of 11,786 binary name features (See **Methods**). These name features are very similar to the molecular fingerprints, providing extra information about the similarities of molecules. Although the random forest model using only the name features has relatively low correlations, the ensemble model aggregating both molecular and name features performs the best, ranking first in predicting the 21 personalized perceptual attributes (**Figure 3C**).

Discriminative chemoinformatic features for olfaction prediction

Our random forest model evaluates the importance of each molecular descriptor in prediction. It is well known that sulfur-containing organic molecules tend to have "garlic" odor, whereas esters are often smelled as "fruity". Many models have been built to correlate molecular size and complexity with the "pleasantness" of a compound. However, it remains unclear what chemical features of a molecule decide its multiple odor attributes. Random forest enables us to estimate the importance of each chemical feature by permuting the values of a feature across samples and computing the increase in prediction error. We calculate the increased delta error of each

chemical feature for all 21 olfactory qualities. Interestingly, top-ranking features used by random forest do not necessarily have high linear Pearson's correlations with observed ratings. For example, the correlation coefficients of the top 5 features for "decayed" prediction are listed in **Supplementary Table 1**. The molecular feature P_VSA_m_4 (interpreted as the presence of sulfur atoms) ranks first and has the largest correlation. However, the 2nd and 3rd features in random forest have nearly no correlations with observed perceptions. Upon inspecting the top 5 features that have the largest correlation values (yellow columns in **Supplementary Table 1)**, we notice that they are all related to sulfur atoms, leading to high redundancy and inter-correlation.

By analyzing all the top 5 features ranked by the delta error, we find that discriminative molecular features are more likely to be low- or non-degenerative. The complete lists of top 20 features ranked by delta error or Pearson's correlation are shown in **Supplementary Tables 2 and 3**, respectively. Interestingly, simple chemical features (molecular weight, number of sulfur atoms, presence of a functional group, etc.) are not very powerful in prediction, because they display high degeneracy – different molecules may have identical or similar values [32–34]. Features with low- or non-degeneracy more likely play an essential role in our random forest model. The frequency of all top 5 molecular features is represented as the size of words in **Figure 4A**. We find that autocorrelation of a topological structure (ATS) and 3D-MoRSE descriptors occur 24 and 23 times, respectively, whereas simple descriptors such as N% (percentage of N atoms), nRCOOR (number of aliphatic esters) and NssO (number of ssO atoms) are used only once (**Figure 4B**).

To understand how the random forest model works, we projected all molecules onto selected important feature spaces (**Figure 4C**). The color of each molecule represents the strength of the perceived rating. For example, ATS1s and ATS2s are the most important features in predicting

the “intensity” of a molecule. They can be interpreted as the combined information of molecule size and the intrinsic state of all atoms. Molecules with large ATS1s and ATS2s values tend to have low intensity (top right green spots in **Figure 4C**, left panel). Another example is the “pleasantness” rating of a molecule, for which SssO (presence of ester or ether) and P_VSA_i_1 (presence of sulfur or iodine atom) are crucial. Clearly, molecules containing sulfur or iodine atom have lower “pleasantness” values (green spots above the dashed line in **Figure 4C**, middle panel). And it is widely known that ester has a characteristic pleasant odor and lower ethers can act as anesthetics, whereas presence of sulfur atom leads to unpleasant “garlic” and “decayed” odor. Therefore, key features of “garlic” odor include MAXDN (presence of ketone or ester) and R3p+ (presence of sulfur atom). Molecules containing sulfur atom are more likely to be “garlicky”, whereas ketones and esters seldom have such smells (red spots above the dashed line in **Figure 4C**, right panel).

Rebuilding the random forest model with different numbers of key features, we find that a small set of chemical features is sufficient for accurate prediction. These top features selected by random forest may have very low linear Pearson’s correlations with perceived qualities, yet they are powerful in discriminating different odorants. This is because the relationship between molecular features and olfactory perception is inherently non-linear. Intriguingly, random forest with only top 5 features achieves similar performance as random forest with all 4,884 features for almost all olfactory qualities (**Figure 5A** and **Supplementary Figure 3**). The only exception is “intensity”, for which top 15 features are adequate. This result indicates that a small set of chemical features are often sufficient to predict the odor of a molecule. We also test the performance of random forest using features ranked by Pearson’s correlation (**Figure 5B**). The predicting power of these features is lower due to collinearity and redundancy. For example, the top 10 features for “garlic” quality are all related to the number of sulfur atoms, although they display very high correlation values (**Supplementary Table 3**).

Deciphering the divergent multi-odor profiles of structural analogs

Structurally similar compounds with distinct odor profiles were observed in triads and a tetrad of molecules. The first example is three furoate esters (**Figure 6A**). If we compare the functional groups of these three molecules, methyl 2-furoate (**1**) and ethyl 2-furoate (**2**) are more similar, while allyl 2-furoate (**3**) has a unique alkenyl group. Intriguingly, the pairwise correlations between them across 21 perceived olfactory qualities reveal that **2** is the odd one in terms of odor. It is clearly shown in the radar chart of selected odor qualities. Compound **2** has intense “sweet”, “acid” and “urinous” characters, whereas 1 and 3 display more “decayed” odor. The second triad of molecules comprises common L-amino acids: alanine (**4**), leucine (**5**) and valine (**6**) (**Figure 6B**). Their odor profiles differ a lot, especially between **4** and **5**. **4** has “fruit”, “sweet” and “flower” odors, whereas **5** is characterized by “sour”, “decayed”, “sweaty” and “intensity”. **6** has a relatively similar odor profile as **4**. The odd one in structural terms is **4** as it has the smallest side chain, whereas the odd one in terms of odor is **5**. The last group of molecules are thiazole (**7**) and its derivatives (**8**-**10**) (**Figure 6C**). **9** stands out because of its signature “grass” odor, whereas both **9** and **10** have very high “chemical” odor.

Our random forest model distinguishes the multi-odor profiles of structural analogs using complex molecular features. Although these analogs are extremely similar in terms of chemical structure and functional group, the values of their 2- and 3-dimensional molecular descriptors are distinct. The average rating of each molecule is represented by its color and the structural analogs mentioned above are shown in a larger size (**Figure 6**, right panels). The top features used by our random forest model clearly separate the structurally similar molecules with dissimilar odor attributes. For example, **10** with strong “grass” odor (top right orange diamond in

Figure 6C, the 3rd panel) has large SaaS and L3m values, whereas **7** and **8** with weak “grass” odor (bottom-right green circle and triangle) have relatively small values. **9** (middle right yellow square) with medium “grass” odor has around average values among the tetrad.

Discussion

The complex and sophisticated signaling of diverse odorants has fascinated scientists for many decades, yet the molecular mechanisms of olfactory perception are still not fully understood. One odorant interacts with a broad range of olfactory receptors and each olfactory receptor recognizes multiple odorants, leading to the complicated tuning of olfactory perception [35,36]. In addition, neuron firing is intrinsically nonlinear in nature, requiring the membrane potential to be raised above threshold. Therefore, a nonlinear random forest model is well-suited to the olfactory prediction and avoids overfitting, given a comparatively small sample size and much larger feature spaces. Moreover, random forest is relatively robust to label noise and outliers [29], considering the vast variability of odor ratings among individuals.

The linguistic descriptions of smells vary among individuals, especially when they lack experience and training [37]. This finding suggests that using a low-variant dataset of odorants rated by professional perfumers may further improve the performance of predictive models. Besides, using semantic descriptors itself introduces biases, and alternative approaches such as perceptual similarity rating of odorants should be considered [23]. Recognizing that extra Morgan-NSPDK features created by matching target molecules against reference odorants increase the predicting performance [27], a larger training set of diverse molecules, including natural odorant products, will be helpful to build more accurate models.

Our random forest model potentially provides an alternative for rational odorant design [38,39]. In addition to modifications of a natural odorant product, the perceptual dataset used in this study consists of many untested molecules, providing new odorant scaffolds of different semantic qualities. Moreover, a small set of top-ranking features estimated by the random forest model is sufficient to accurately predict human olfactory perception, largely reducing the input

feature spaces. This model is potentially useful for evaluation of new molecules, and modification of these discriminative features provides an alternative for rational odorant design. Like the association of functional groups with certain odors, this study may link complex chemoinformatic features to a broader range of odors, providing a useful perspective for understanding olfactory perception mechanisms.

Methods

Pre-processing of the dataset

There were many cases where subjects indicated that they smelled nothing so the intensity rating was automatically set to “0” and the ratings for other perceptual attributes were left blank (NaN); therefore, we have removed all the ‘NaN’ entries. For the intensity attribute, we used the target values at “1/1,000” dilution and all replicates were treated as separate examples. For pleasantness and 19 semantic attributes, we used target values at 'high' concentration as a set of examples, and the average value at both 'high' and 'low' concentrations as another set of examples, thus doubling the training set. The input molecular features were scaled to values between 0 and 1. The scaling formula is given as:

$$x' = \frac{x - \min(x)}{\max(x) - \min(x)}$$

where x is the original value and x’ is the scaled value.

Selection of base learner

To address the large variability of perceived odor qualities among individuals, we tried a range of different machine learning algorithms (linear, ridge, SVM with rbf kernel and random forest) to find the best performing base learner. We applied a 5-fold cross-validation to the training data (407 molecules) and evaluated the performance based on the correlations of the 21 perceptual attributes between the predicted and observed ratings. Random forest outperformed other base learners and was used in the follow-up improvement of our model.

Integrating individual and population ratings

The perceptual rating of attributes varies greatly. To reduce the effects of noise and outliers, we introduce a weighting factor, α, as the weight for individual ratings and (1 – α) as the weight for population ratings. The re-weighted target value y is given as:

$$y = \alpha \times y_{individual} + (1 - \alpha) \times y_{population}$$

where $y_{individual}$ is the rating from an individual and $y_{population}$ is the average rating from 49 individuals. Different values of α were tested and evaluated by the correlation of 21 perceptual attributes. α = 0.2 had the best performance and was used in our final model.

Creating name features of molecules

In the past, sliding window-based (overlapping patterns) strategies were applied successfully to develop residue level predictions [40,41]. We used a sliding window of 4-letter size to extract features from the molecule names. For example, 4-letter indexing generated a total of 7 sliding windows from “acetic acid” (ACET, CETI, ETIC, TIC_, IC_A, _ACI, ACID). We created 11786 binary name features from all molecule names using this sliding window approach. If a window pattern is present in the molecule name, ‘1’ was assigned to that feature, otherwise ‘0’ was used while creating input name features.

Evaluation of the importance of each feature by random forest

The importance of each feature was evaluated by permuting the values across observations and computing the increase in prediction error by random forest. The increased delta error of

each chemical feature for all 21 olfactory attributes was calculated and ranked. Larger delta error implies that the feature is more important and discriminative in prediction.

Figure legends

Figure 1. The overview of the olfaction prediction

The observed perceptions form a 3-dimensional array, where the 3 dimensions are 476 molecules, 49 individuals and 21 olfactory attributes. The input chemoinformatic features form a 2-dimensional matrix, where the rows are 476 molecules and columns are 4884 molecular descriptors. Our random forest model is built on the training set (407 molecules) and the individual responses for the test set (69 molecules) are predicted. The final evaluation is based on the Pearson's correlation between observed and predicted perceptions.

Figure 2. Variability of olfactory perception among individuals

A. The intensity ratings for all molecules at low and high concentrations from individuals 10, 29 and 46. Blue lines represent the ideal cases, in which the rating values increase as the concentration becomes higher. Conversely, red lines represent decreased rating values at high concentration. **B.** The density distributions of the intensity ratings from these three individuals. Blue lines are the fitting curves of the density distribution. The intensity ratings and density distributions from all individuals are shown in **Supplementary Figures 1 and 2**, respectively. **C.** The "garlic" and "warm" rating distributions among 49 individuals for 2-acetylpyridine and cyclopentanethiol, respectively. **D.** The coefficients of variation of 21 perceptual attributes in the increasing order.

Figure 3. The performance of different models and strategies

From left to right, the Pearson's correlation coefficients of intensity, pleasantness and 19 semantic descriptors from 5-fold cross-validations are shown as boxplot. The red base-learners or strategies are used in our final model. **A.** The performance of four different base-learners: linear, ridge, SVM and random forest. **B.** The performance of using different values of weighting

factor α. **C.** The performance of using molecular feature alone, name feature alone, and both molecular and name features.

Figure 4. Top discriminative features used in random forest

A. The word cloud of top 5 features used in predicting 21 perceptual attributes. **B.** The pie chart of molecular descriptor categories in top 5 features. **C.** Projection of all molecules onto selected discriminative feature spaces. The color of each spot represents the relative strength of the perceived rating, averaged among 49 individuals. The dashed lines display the possible decision boundaries created by random forest.

Figure 5. The performance of random forest using top features

From left to right, the Pearson's correlation coefficients of intensity, pleasantness and 19 semantic descriptors from 5-fold cross-validations are shown as boxplots. The red model is the random forest using all chemoinformatic features. **A.** The performance of random forest using top 5, 10, 15, 20 features ranked by delta error. **B.** The performance of random forest using top 5, 10, 15, 20 features ranked by Pearson's correlation.

Figure 6. Distinguishing different odor profiles of structurally similar molecules by random forest

The odor profiles of **A. 3** furoate esters, **B.** 3 amino acids and **C.** 4 thiazole derivatives. The left panel shows the pairwise correlations between structurally similar molecules. The color of each edge represents the correlation value across 21 perceptual attributes. The middle panel shows the radar charts of selected odor attributes. The symbol and color correspond to the molecule on the left. The right panel displays the projections of all molecules onto selected discriminative feature spaces. The color of each spot represents the relative strength of the perceived rating, averaged among 49 individuals. The larger symbols correspond to the molecules on the left.

These structural analogs with different odors are clearly separated in the 2-dimensional feature spaces.

Availability of supporting data

The DREAM olfaction challenge dataset is available at:

https://www.synapse.org/ - !Synapse:syn2811262/wiki/78368

The model details and source codes are available at:

https://www.synapse.org/ - !Synapse:syn3354800/wiki/231431

Completing interests

The authors declare that they have no competing interests.

Authors' contributions

YG conceived and designed the prediction algorithm. YG and HL performed computational analysis of the observed and predicted data. HL analyzed the discriminative chemoinformatic features and prepared figures. HL, BP, GO and YG contributed to the writing of the manuscript. All authors read and approved the final manuscript.

Acknowledgements

This work is supported by NSF 1452656 and Alzheimer's Association BAND-15-367116 [Biomarkers Across Neurodegenerative Diseases Grant 2016].

Reference

1. Gaillard I, Rouquier S, Giorgi D. Olfactory receptors. Cell. Mol. Life Sci. 2004;61:456–69.

2. Buck LB. Olfactory receptors and odor coding in mammals. Nutr. Rev. 2004;62:S184-NaN-S241.

3. Read JCA. The place of human psychophysics in modern neuroscience. Neuroscience [Internet]. IBRO; 2015;296:116–29. Available from: http://dx.doi.org/10.1016/j.neuroscience.2014.05.036

4. Sell CS. On the unpredictability of odor. Angew. Chemie - Int. Ed. 2006;45:6254–61.

5. Laska M, Teubner P. Olfactory discrimination ability for homologous series of aliphatic alcohols and aldehydes. Chem. Senses. 1999;24:263–70.

6. Boesveldt S, Olsson MJ, Lundström JN. Carbon chain length and the stimulus problem in olfaction. Behav. Brain Res. [Internet]. Elsevier B.V.; 2010;215:110–3. Available from: http://dx.doi.org/10.1016/j.bbr.2010.07.007

7. Keller A, Zhuang H, Chi Q, Vosshall LB, Matsunami H, Al. E. Genetic variation in a human odorant receptor alters odour perception. Nature. 2007;449:468–72.

8. Chastrette M. Trends in Structure-Odor Relationship. SAR QSAR Environ. Res. 1997;6:215–54.

9. Boelens H. Structure—activity relationships in chemoreception by human olfaction. Trends Pharmacol. Sci. [Internet]. Elsevier Current Trends; 1983;4:421–6. Available from: http://linkinghub.elsevier.com/retrieve/pii/0165614783904753

10. Edwards PA, Jurs PC. Correlation of odor intensities with structural properties of odorants. Chem. Senses. Oxford University Press; 1989;14:281–91.

11. Mamlouk AM, Chee-Ruiter C, Hofmann UG, Bower JM. Quantifying olfactory perception: mapping olfactory perception space by using multidimensional scaling and self-organizing maps. Neurocomputing. 2003;52:591–7.

12. Zarzo M, Stanton DT. Identification of Latent Variables in a Semantic Odor Profile Database Using Principal Component Analysis. Chem. Senses. 2006;31:713–24.

13. Mainland JD, Lundström JN, Reisert J, Lowe G. From molecule to mind: an integrative perspective on odor intensity. Trends Neurosci. 2014;37:443–54.

14. Kermen F, Chakirian A, Sezille C, Joussain P, Le Goff G, Ziessel A, et al. Molecular complexity determines the number of olfactory notes and the pleasantness of smells. Sci. Rep. 2011;1:206.

15. Zarzo M. Hedonic Judgments of Chemical Compounds Are Correlated with Molecular Size. Sensors. 2011;11:3667–86.

16. Khan RM, Luk C-H, Flinker A, Aggarwal A, Lapid H, Haddad R, et al. Predicting Odor Pleasantness from Odorant Structure: Pleasantness as a Reflection of the Physical World. J. Neurosci. [Internet]. 2007;27:10015–23. Available from: http://www.jneurosci.org/cgi/doi/10.1523/JNEUROSCI.1158-07.2007

17. Menashe I, Man O, Lancet D, Gilad Y. Different noses for different people. Nat. Genet. 2003;34:143–4.

18. Keydar I, Ben-Asher E, Feldmesser E, Nativ N, Oshimoto A, Restrepo D, et al. General Olfactory Sensitivity Database (GOSdb): Candidate Genes and their Genomic Variations. Hum. Mutat. 2013;34:32–41.

19. Perez M, Nowotny T, d'Ettorre P, Giurfa M. Olfactory experience shapes the evaluation of odour similarity in ants: a behavioural and computational analysis. Proc. R. Soc. B Biol. Sci. 2016;283:20160551.

20. Haddad R, Khan R, Takahashi YK, Mori K, Harel D, Sobel N. A metric for odorant comparison. Nat. Methods. 2008;5:425–9.

21. Koulakov AA, Kolterman BE, Enikolopov AG, Rinberg D. In search of the structure of human olfactory space. Front. Syst. Neurosci. 2011;5:65.

22. Castro JB, Ramanathan A, Chennubhotla CS. Categorical Dimensions of Human Odor

Descriptor Space Revealed by Non-Negative Matrix Factorization. Schaefer A, editor. PLoS One. 2013;8:e73289.

23. Snitz K, Yablonka A, Weiss T, Frumin I, Khan RM, Sobel N. Predicting Odor Perceptual Similarity from Odor Structure. Diedrichsen J, editor. PLoS Comput. Biol. [Internet]. 2013;9:e1003184. Available from: http://dx.plos.org/10.1371/journal.pcbi.1003184

24. Dravnieks A. Odor quality: semantically generated multidimensional profiles are stable. Science. 1982;218:799–801.

25. Dudek AZ, Arodz T, Gálvez J. Computational methods in developing quantitative structure-activity relationships (QSAR): a review. Comb. Chem. High Throughput Screen. [Internet]. 2006;9:213–28. Available from: http://www.ncbi.nlm.nih.gov/pubmed/16533155

26. Nantasenamat C, Isarankura-Na-Ayudhya C, Prachayasittikul V. Advances in computational methods to predict the biological activity of compounds. Expert Opin. Drug Discov. [Internet]. 2010;5:633–54. Available from: http://www.ncbi.nlm.nih.gov/pubmed/22823204

27. Keller A, Gerkin RC, Guan Y, Dhurandhar A, Turu G, Szalai B, et al. Predicting human olfactory perception from chemical features of odor molecules. Science (80-.). [Internet]. 2017;355:820–6. Available from: http://www.sciencemag.org/lookup/doi/10.1126/science.aal2014

28. Saez-Rodriguez J, Costello JC, Friend SH, Kellen MR, Mangravite L, Meyer P, et al. Crowdsourcing biomedical research: leveraging communities as innovation engines. Nat. Rev. Genet. 2016;17:470–86.

29. Breiman L. Randomforest2001. 2001;1–33.

30. Keller A, Vosshall LB. Olfactory perception of chemically diverse molecules. BMC Neurosci. 2016;17:55.

31. Todeschini R, Consonni V, editors. Molecular Descriptors for Chemoinformatics [Internet]. Weinheim, Germany: Wiley-VCH Verlag GmbH & Co. KGaA; 2009. Available from: http://doi.wiley.com/10.1002/9783527628766

32. Godden JW, Bajorath J. Shannon entropy--a novel concept in molecular descriptor and diversity analysis. J. Mol. Graph. Model. [Internet]. 2000;18:73–6. Available from: http://www.ncbi.nlm.nih.gov/pubmed/10935210

33. Godden JW, Bajorath J. Chemical descriptors with distinct levels of information content and varying sensitivity to differences between selected compound databases identified by SE-DSE analysis. J. Chem. Inf. Comput. Sci. [Internet]. 2002;42:87–93. Available from: http://www.ncbi.nlm.nih.gov/pubmed/11855971

34. Godden JW, Bajorath J. An Information-Theoretic Approach to Descriptor Selection for Database Profiling and QSAR Modeling. QSAR Comb. Sci. [Internet]. 2003;22:487–97. Available from: http://doi.wiley.com/10.1002/qsar.200310001

35. Zhao H. Functional Expression of a Mammalian Odorant Receptor. Science (80-.). 1998;279:237–42.

36. Malnic B, Hirono J, Sato T, Buck LB. Combinatorial receptor codes for odors. Cell [Internet]. 1999;96:713–23. Available from: http://www.ncbi.nlm.nih.gov/pubmed/10089886

37. Livermore A, Laing DG. Influence of training and experience on the perception of multicomponent odor mixtures. J. Exp. Psychol. Hum. Percept. Perform. [Internet]. 1996;22:267–77. Available from: http://www.ncbi.nlm.nih.gov/pubmed/8934843

38. Sell C. Structure-odor relations: a modern perspective. 2008;

39. Turin L. Chemistry and Technology of Flavors and Fragrances [Internet]. Rowe DJ, editor. Oxford, UK: Blackwell Publishing Ltd.; 2004. Available from: http://doi.wiley.com/10.1002/9781444305517

40. Panwar B, Gupta S, Raghava GP. Prediction of vitamin interacting residues in a vitamin binding protein using evolutionary information. BMC Bioinformatics. 2013;14:44.

41. Panwar B, Raghava GP. Prediction of uridine modifications in tRNA sequences. BMC Bioinformatics. 2014;15:326.

Supplementary data

Supplementary Table 1. The top 5 features ranked by random forest delta error or Pearson's correlation

The blue columns are the top 5 features ranked by delta error. Their corresponding correlations are also provided. The autocorrelation features "GATS2e" and "GATS2s" have almost zero correlations. The yellow columns are the top 5 features ranked by Pearson's correlation. Although these features have relatively large correlation values, they are all related to sulfur atom(s), leading to high redundancy and inter-correlation.

	Top 5 features by RF delta error			Top 5 features by correlation	
Ranking	Feature	Delta Error	Correlation	Feature	Correlation
1	P_VSA_m_4	0.56	0.48	P_VSA_m_4	0.48
2	GATS2e	0.52	-0.01	nS	0.47
3	GATS2s	0.38	0.02	F01[C-S]	0.46
4	DISPp	0.37	0.21	B01[C-S]	0.46
5	P_VSA_MR_8	0.36	0.39	NssS	0.45

Supplementary Table 2. The top 20 features of 21 perceptual attributes ranked by random forest delta error

(See the extra file: Supplementary_Table2.xlsx)

Supplementary Table 3. The top 20 features of 21 perceptual attributes ranked by Pearson's correlation

(See the extra file: Supplementary_Table3.xlsx)

Supplementary Figure 1. The intensity ratings for all molecules at low and high concentrations from 49 individuals.

Blue lines represent the ideal cases, in which the rating values increase as the concentration becomes higher. Conversely, red lines represent decreased rating values at high concentration.

Supplementary Figure 2. The density distributions of the intensity ratings for all molecules from 49 individuals.

Blue lines are the fitting curves of the density distribution.

Supplementary Figure 3. The performance of random forest using top features ranked by delta error

The Pearson's correlation coefficients of 21 olfactory attributes from 5-fold cross-validations are shown in boxplot. From left to right, the four blue models are random forest using top 5, 10, 15, 20 features. The red model is the random forest using all chemoinformatic features.

Click here to download Figure Figure1.tif

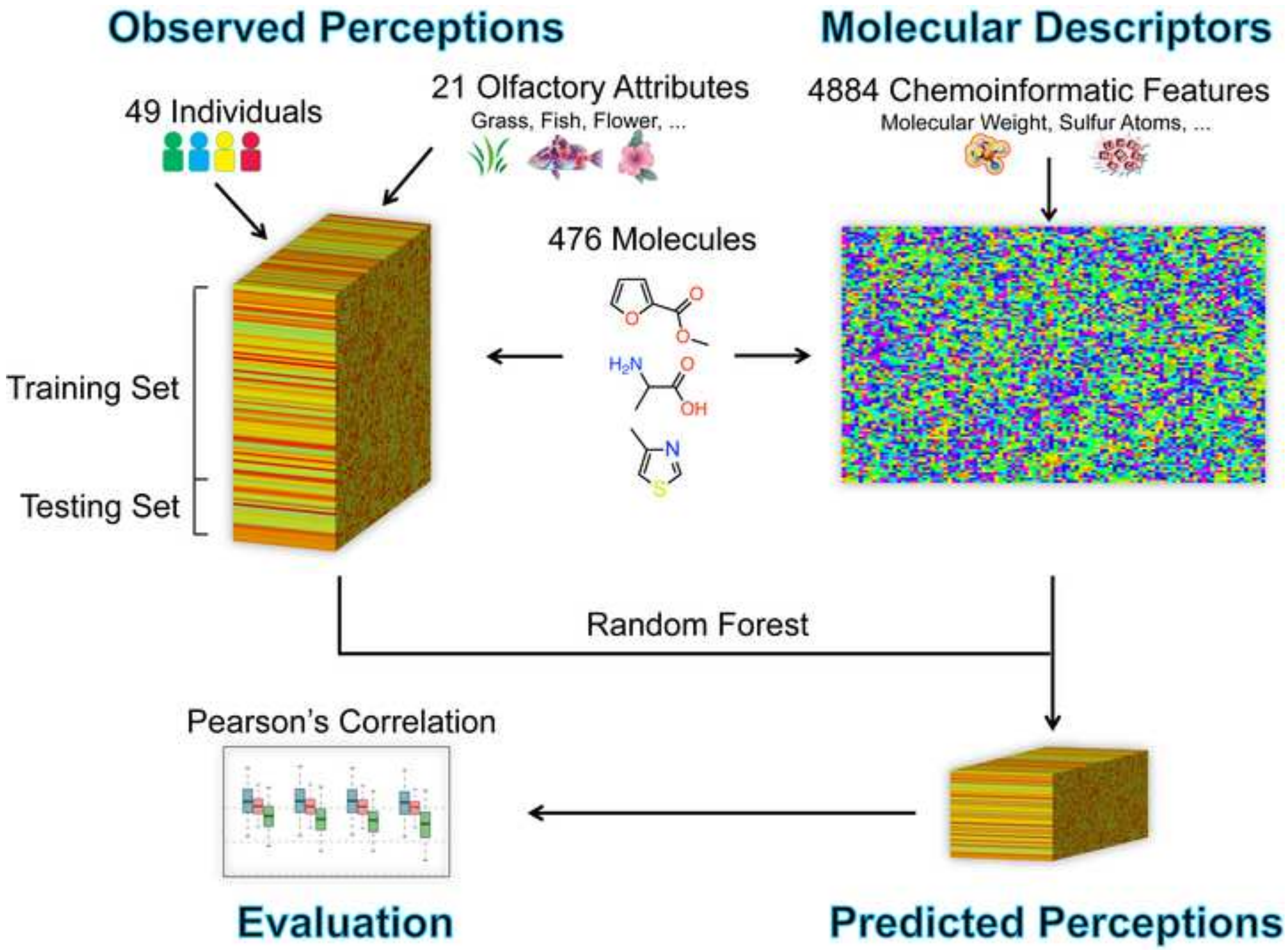

Click here to download Figure Figure2.tif

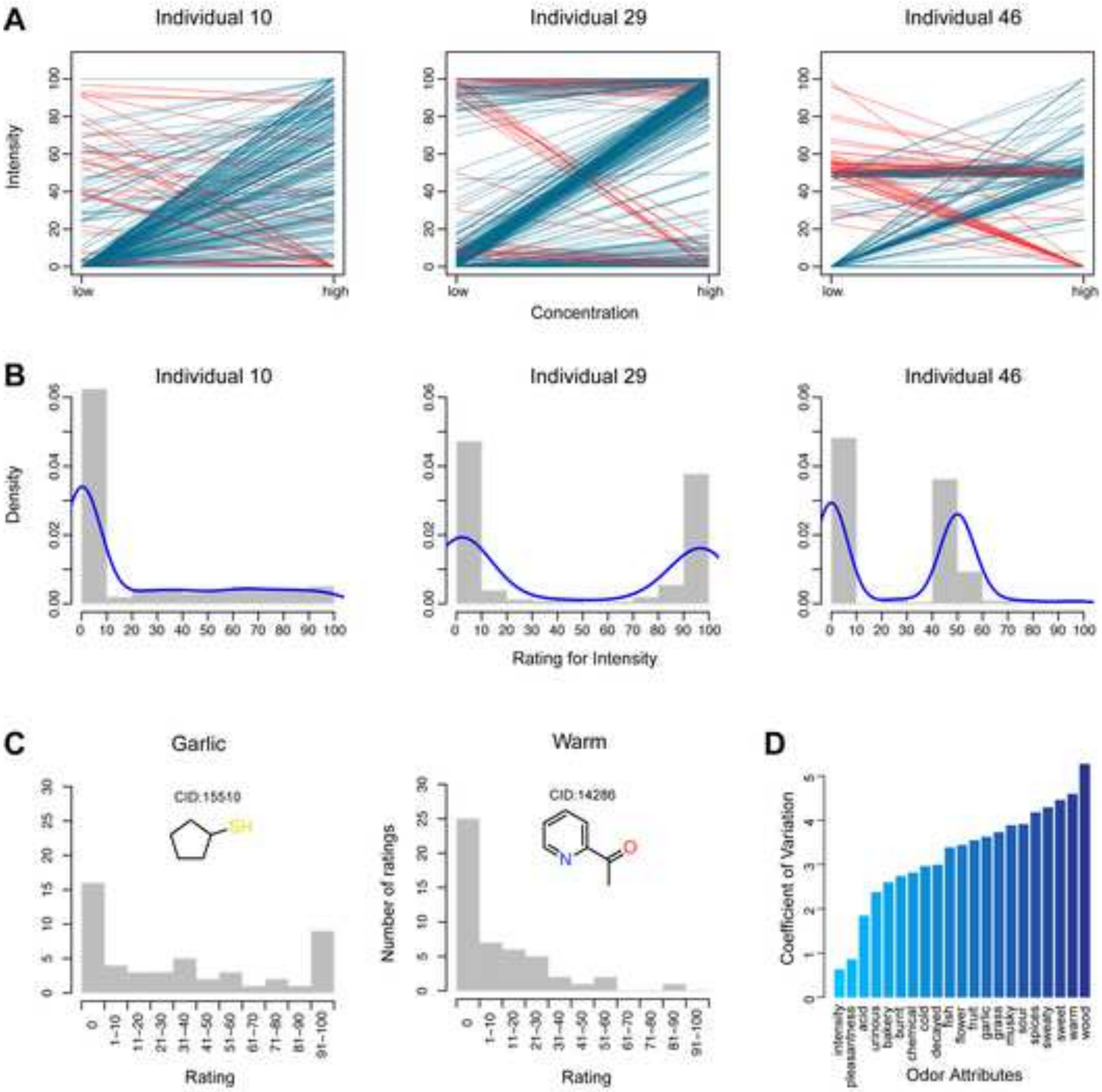

Click here to download Figure Figure3.tif

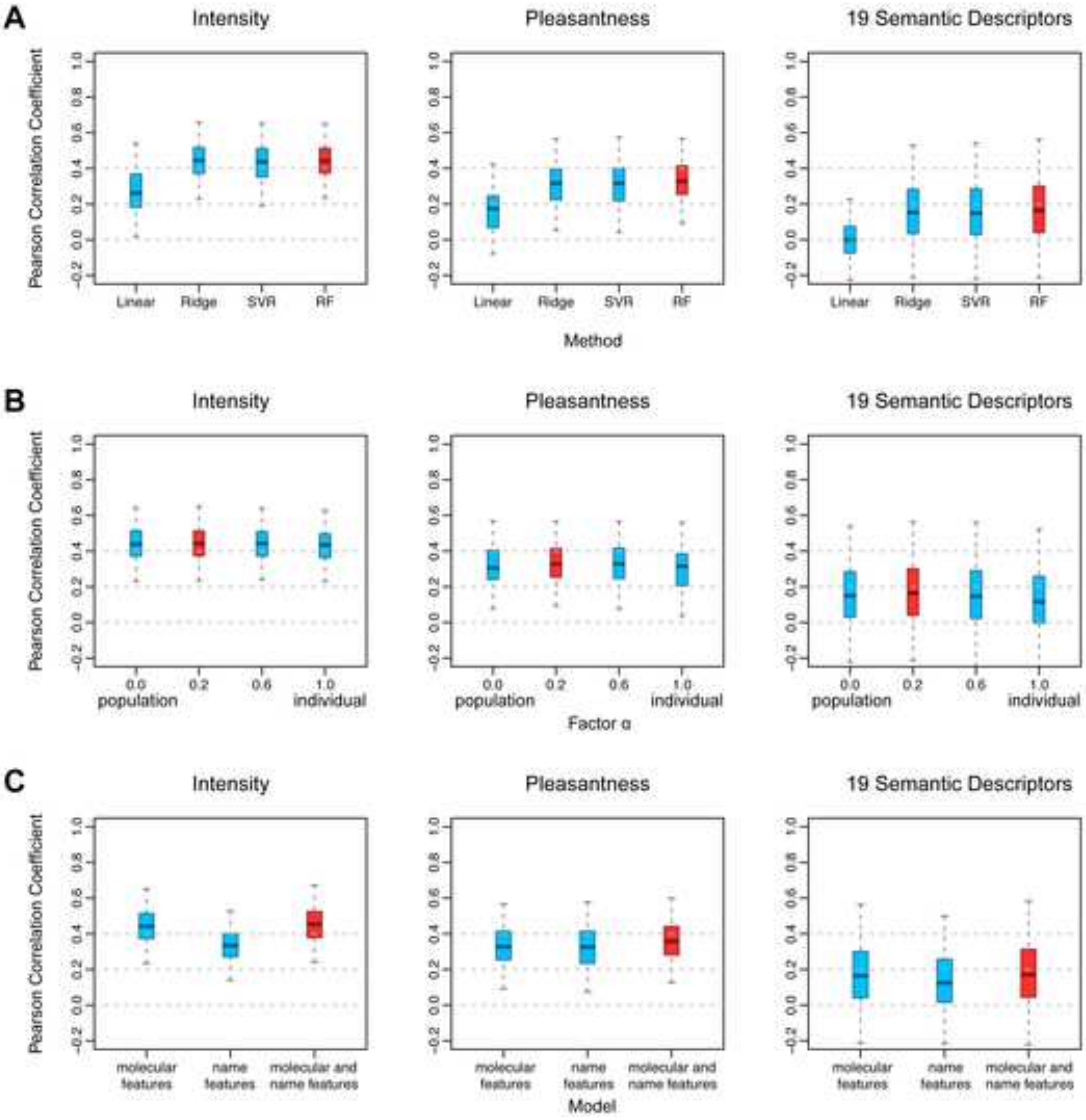

Click here to download Figure Figure4.tif

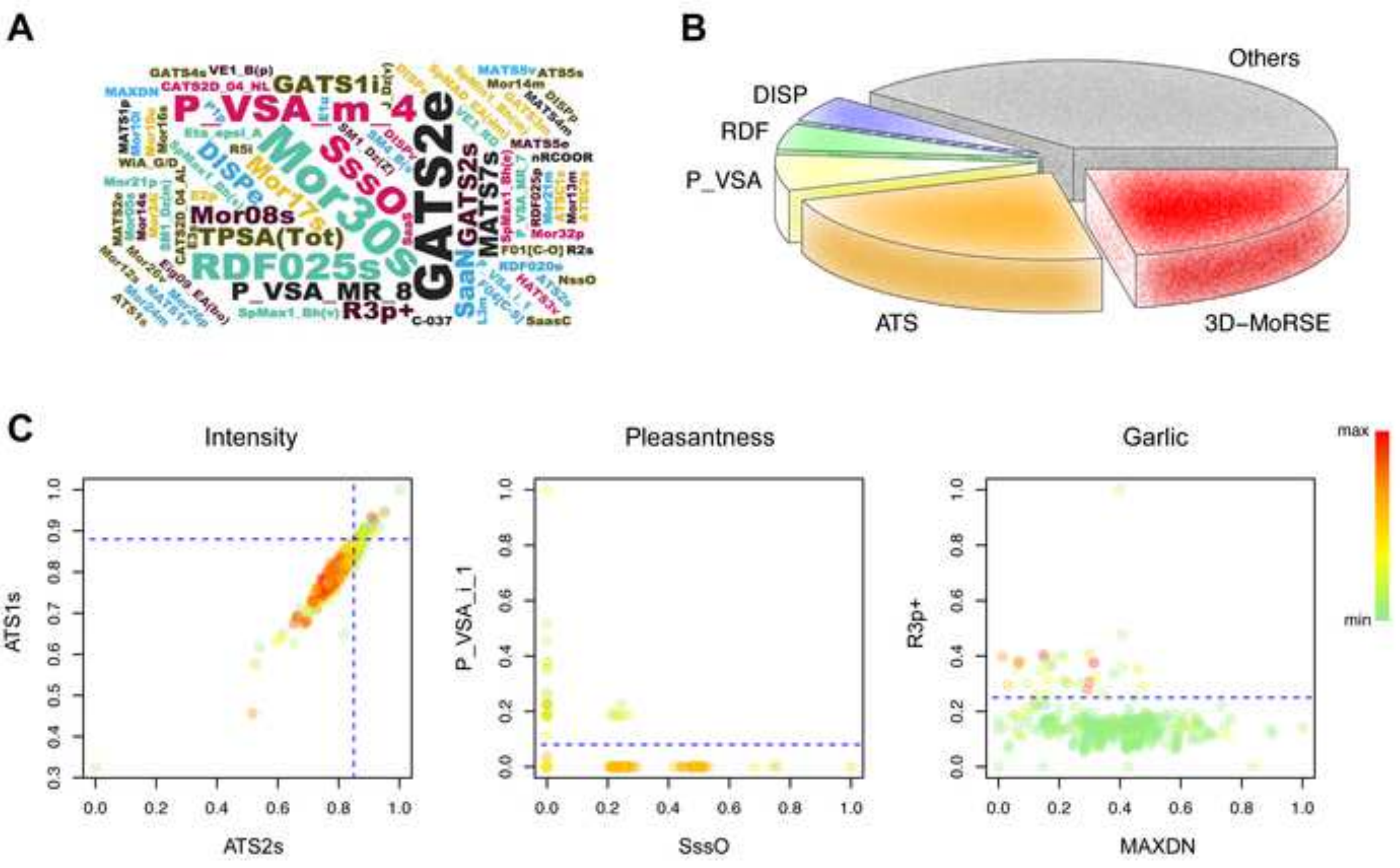

Click here to download Figure Figure5.tif

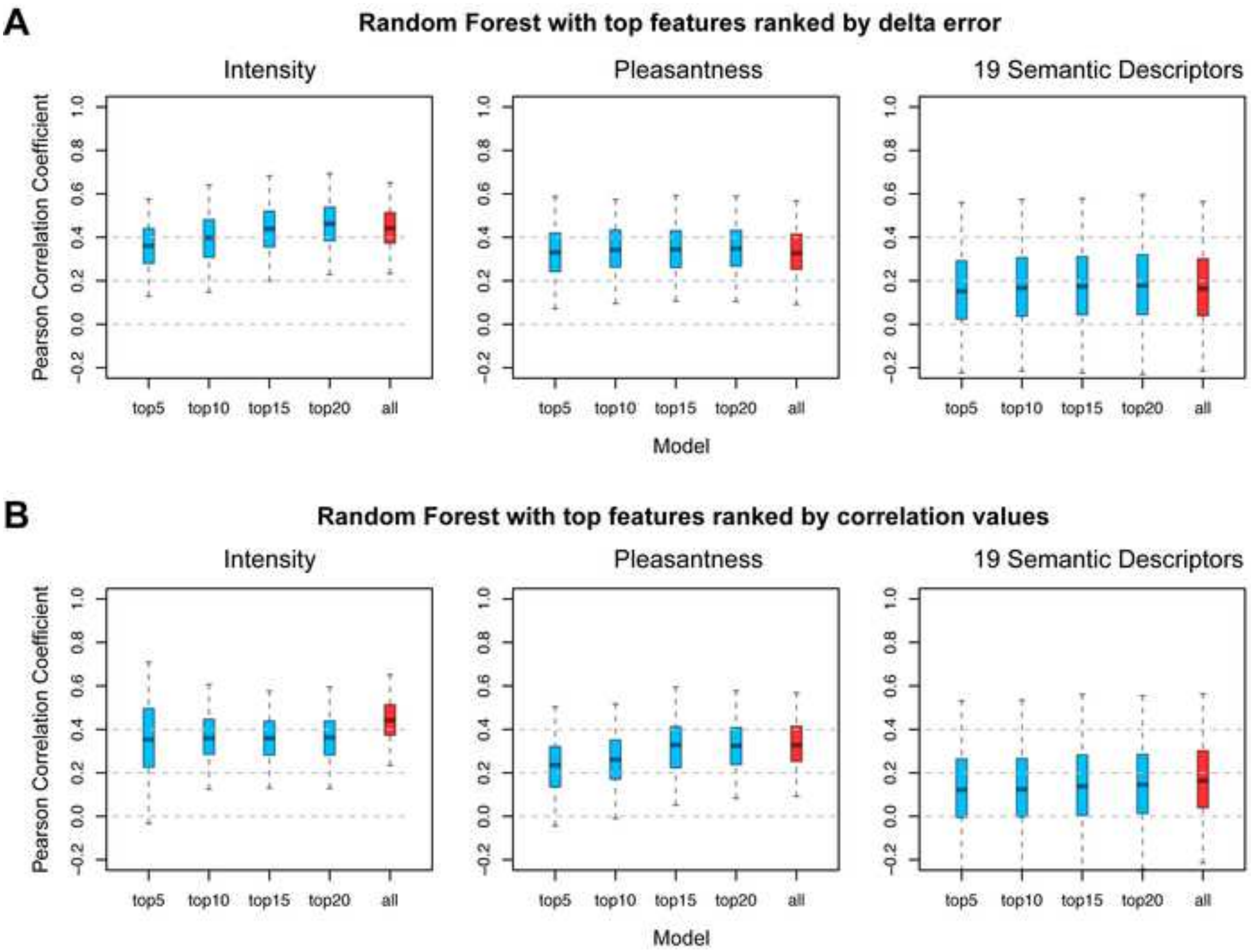

Click here to download Figure Figure6.tif

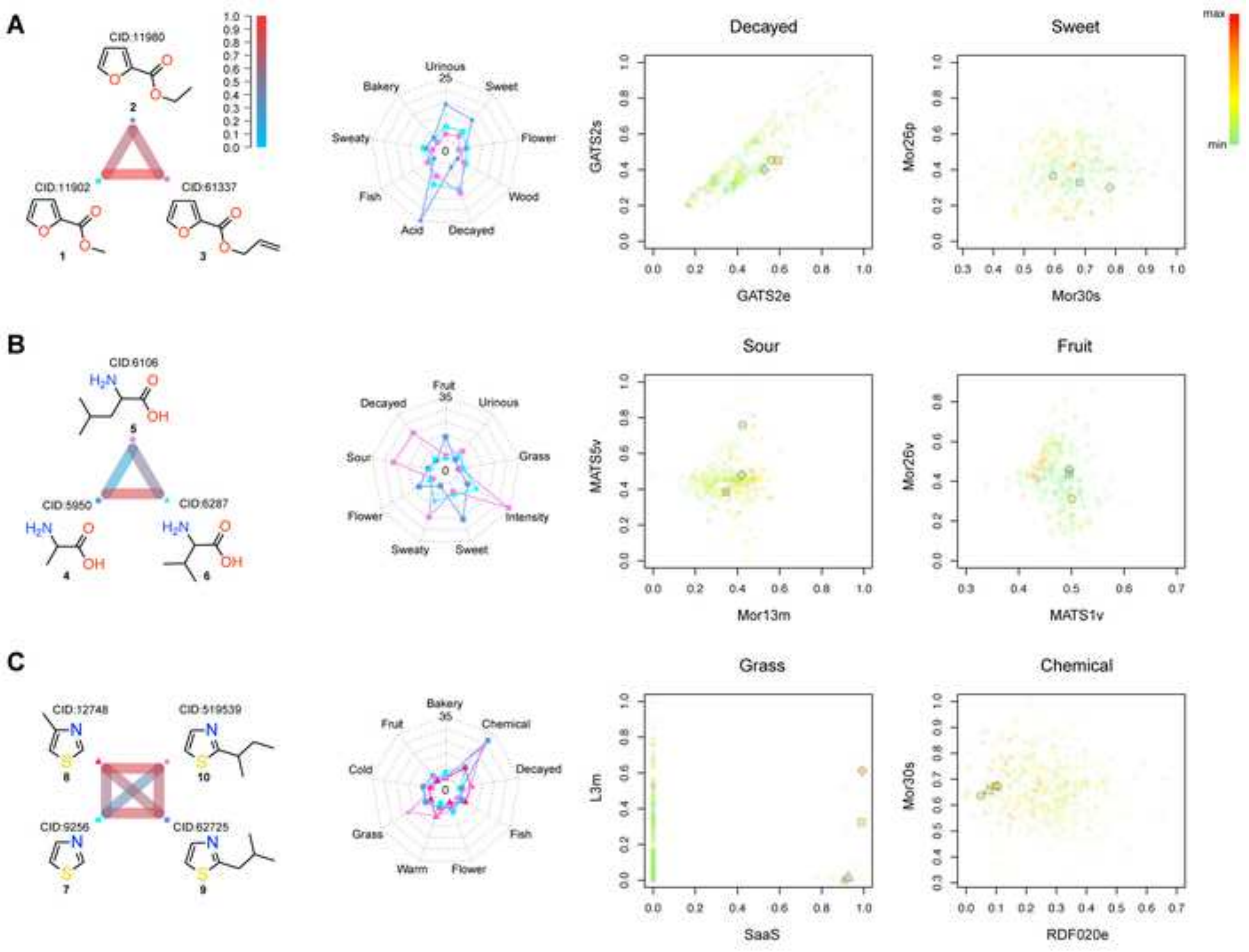

Click here to access/download
Supplementary Material
Supplemenatry_Figure1.tif

Click here to access/download
Supplementary Material
Supplemenatry_Figure2.tif

Click here to access/download
Supplementary Material
Supplemenatry_Figure3.tif

Click here to access/download
Supplementary Material
Supplementary_Table2.xlsx

Click here to access/download
Supplementary Material
Supplementary_Table3.xlsx
