# Supplementary figures and images for: Accurate prediction of personalized olfactory perception from large-scale chemoinformatic features
Source: Gigascience. 2017 Dec 15;7(2):1–11. doi: 10.1093/gigascience/gix127 (PMC5824779; doi:10.1093/gigascience/gix127)

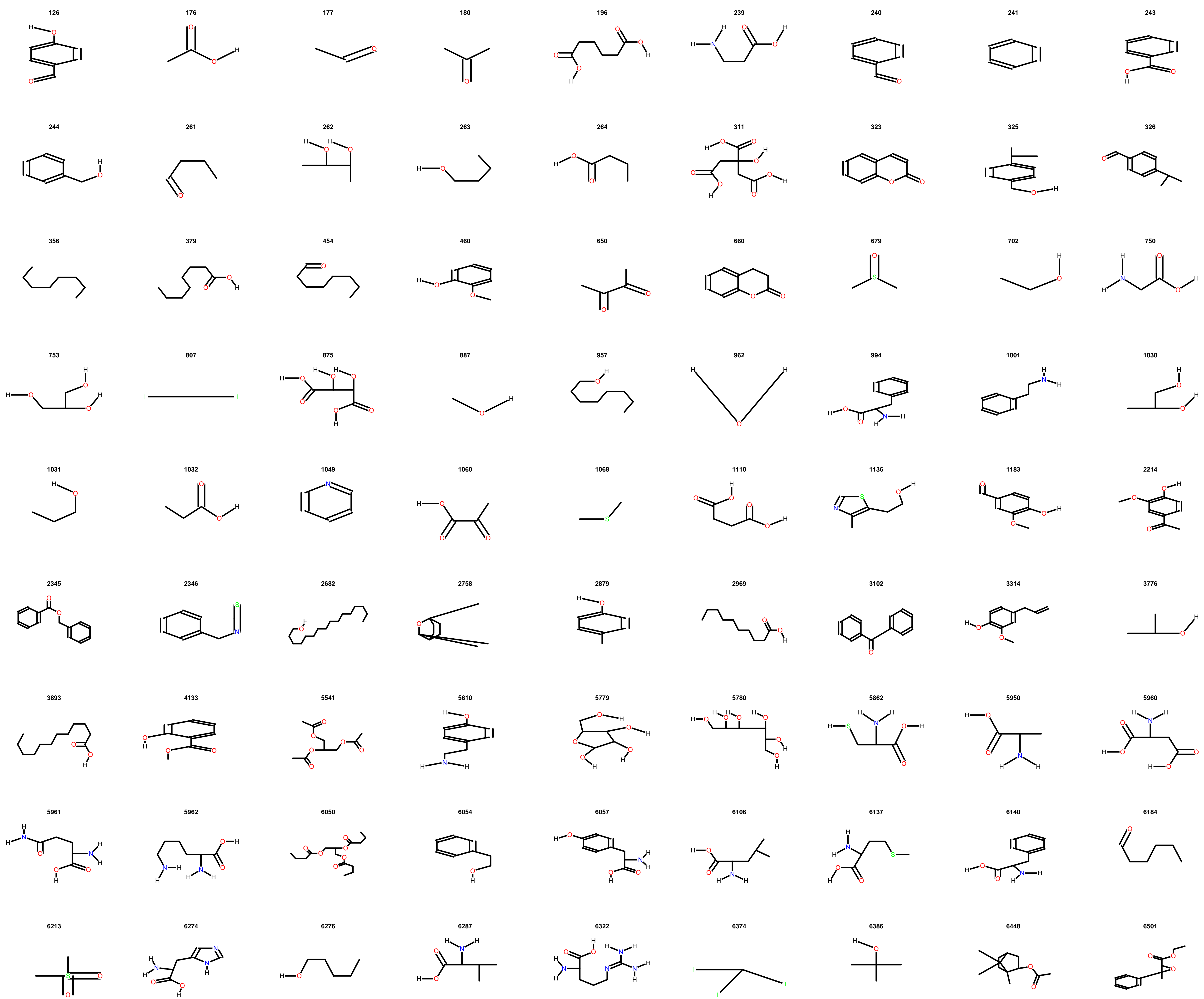

6505

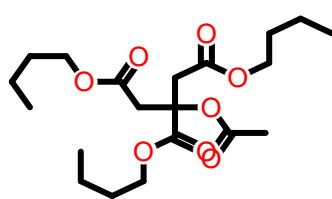

6506

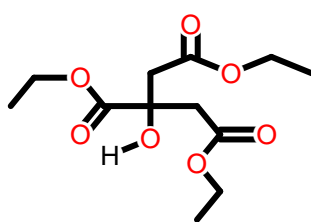

6544

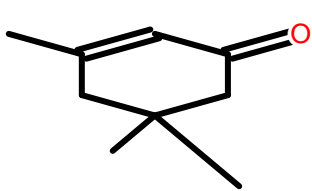

6549

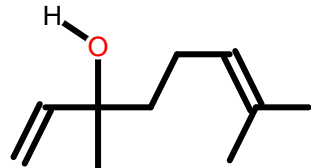

6560

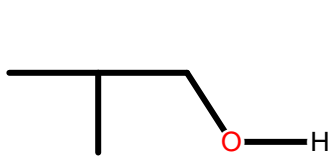

6561

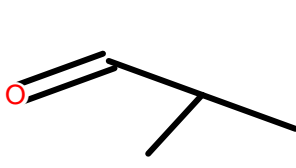

6569

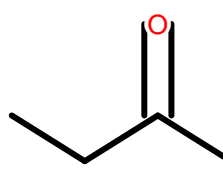

6584

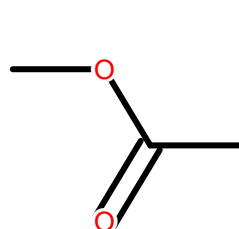

6590

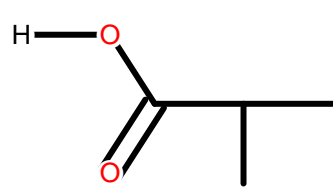

6658

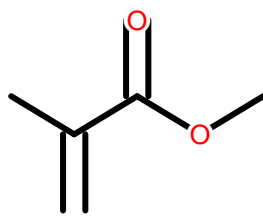

6669

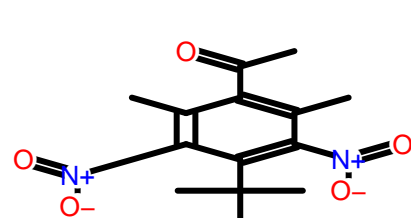

6753

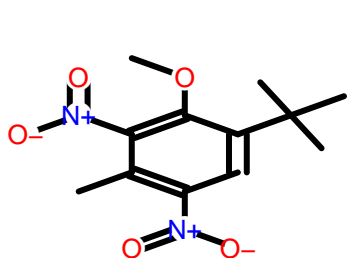

6826

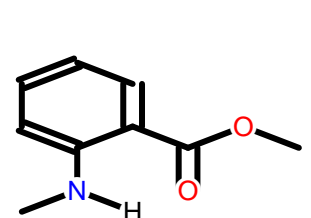

6920

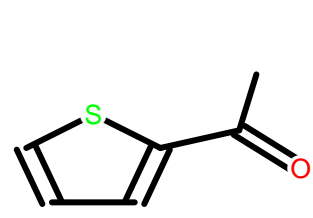

6943

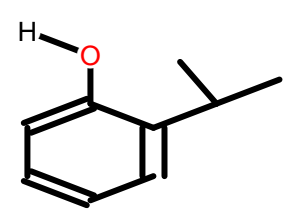

6989

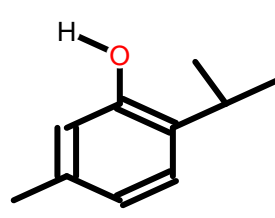

6997

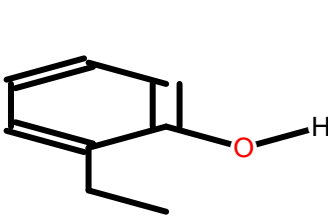

6998

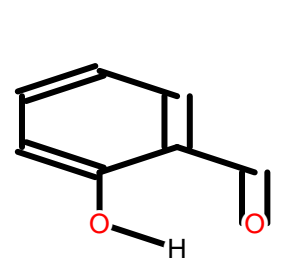

7047

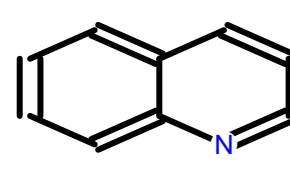

7059

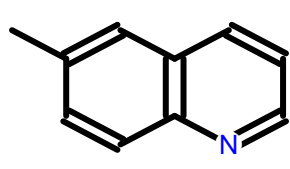

7092

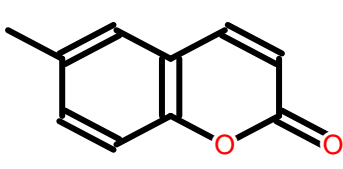

7095

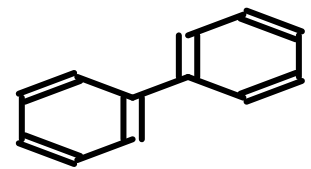

7119

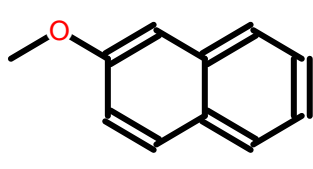

7122

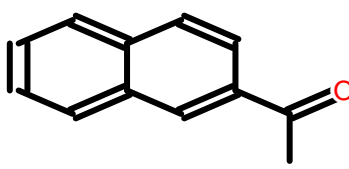

7127

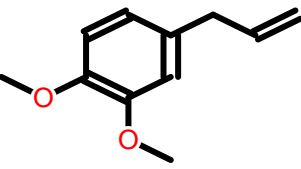

7136

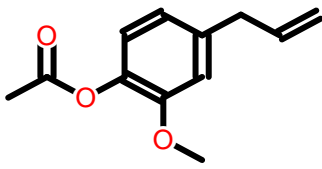

7137

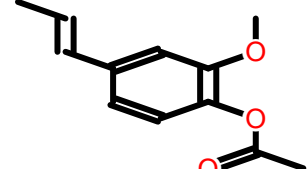

7144

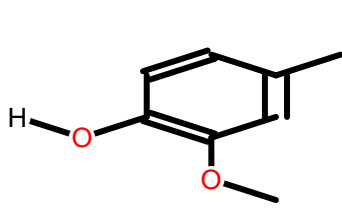

7147

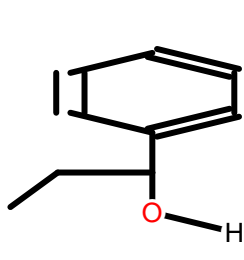

7150

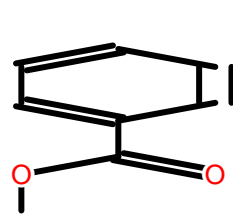

7151

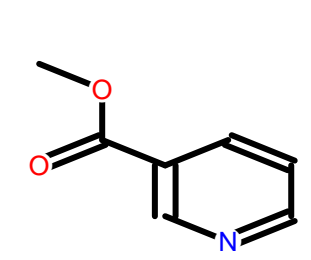

7165

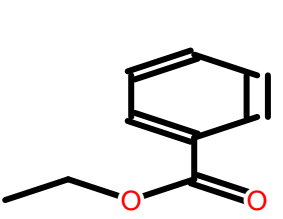

7194

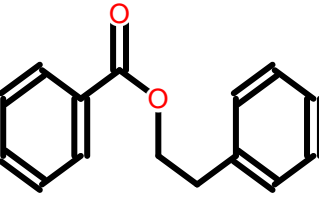

7288

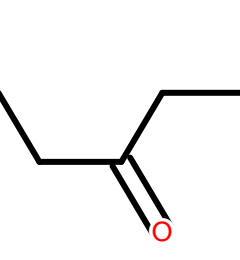

7302

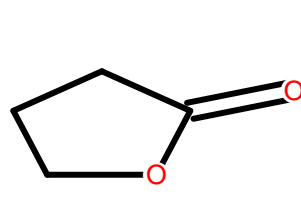

7335

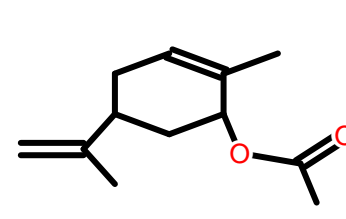

7341

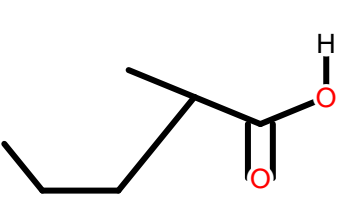

7360

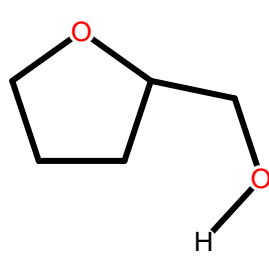

7361

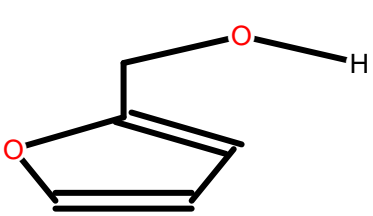

7409

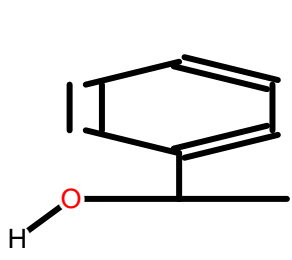

7410

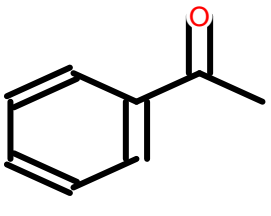

7463

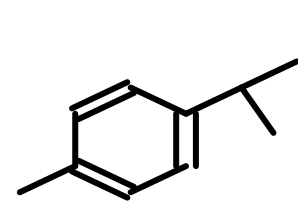

7476

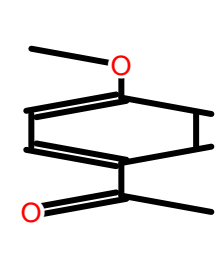

7500

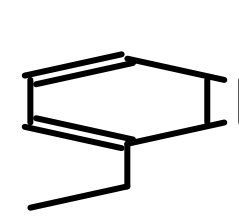

7519

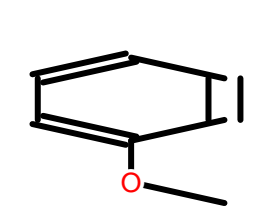

7559

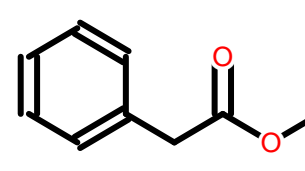

7583

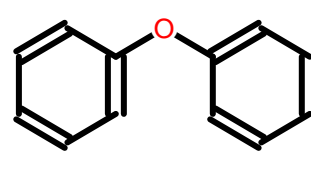

7593

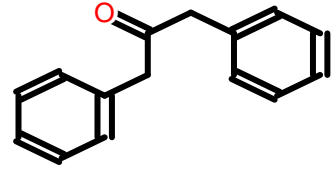

7601

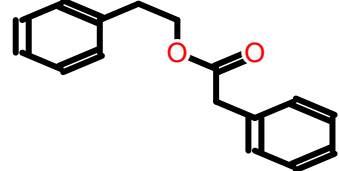

7632

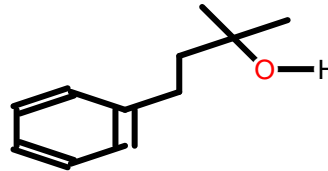

7635

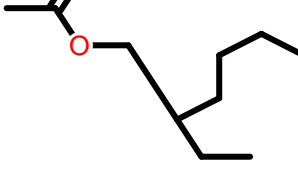

7654

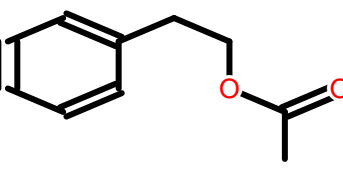

7657

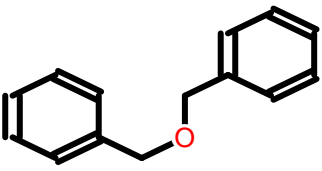

7695

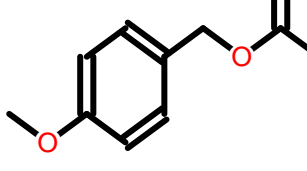

7720

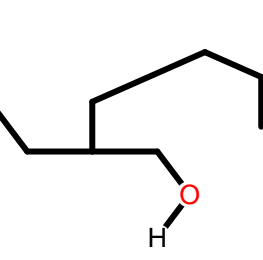

7731

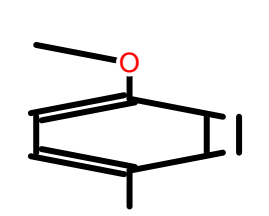

7749

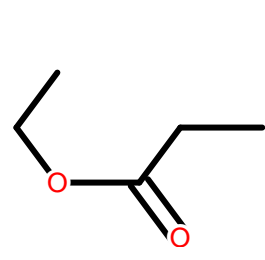

7761

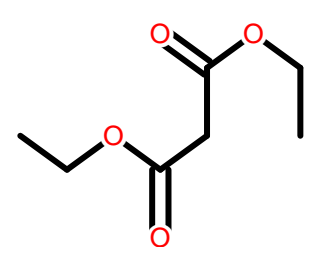

7762

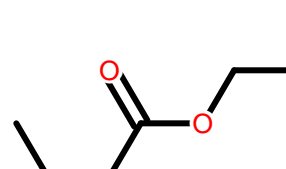

7765

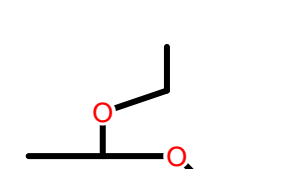

7768

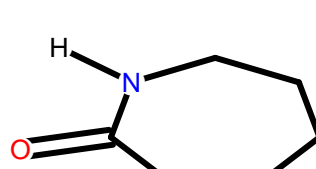

7770

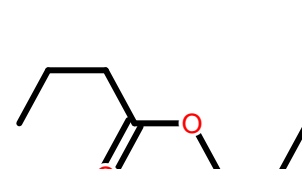

7792

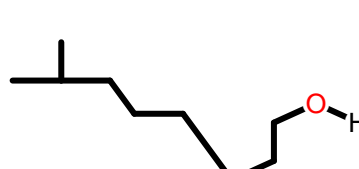

7793

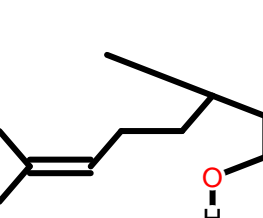

7795

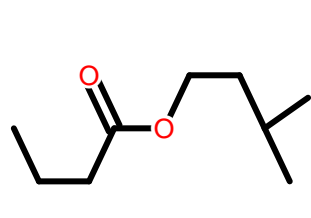

7797

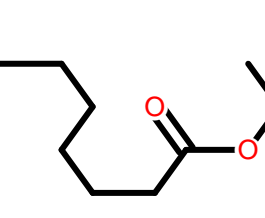

7799

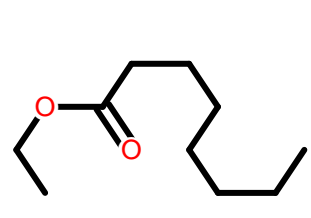

7803

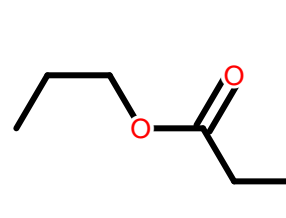

7820

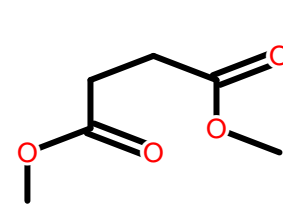

7824

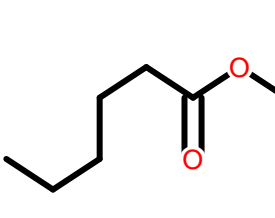

7826

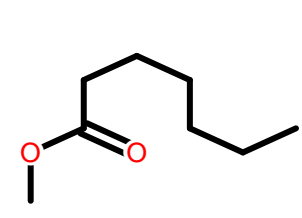

7894

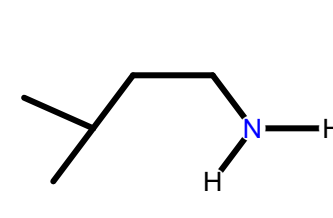

7915

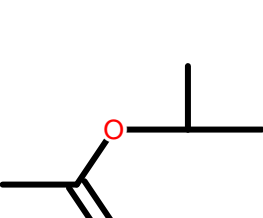

7916

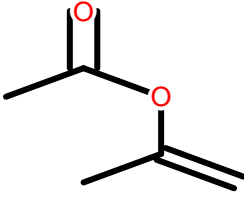

7921

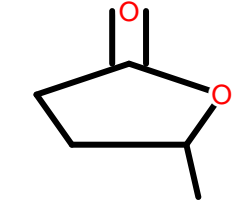

7937

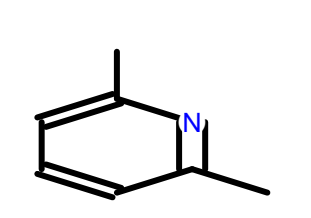

7967

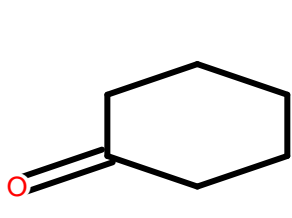

7969

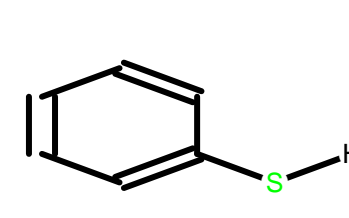

7983

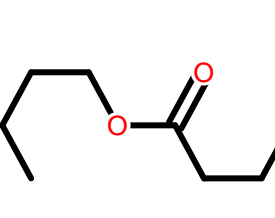

7991

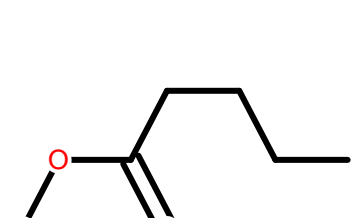

7997

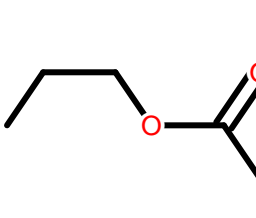

8007

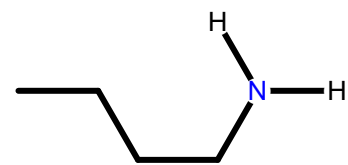

8025

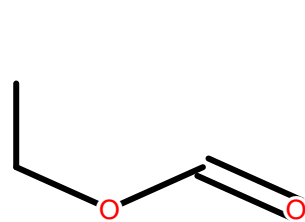

8030

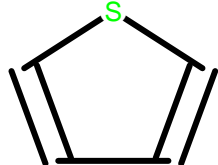

8038

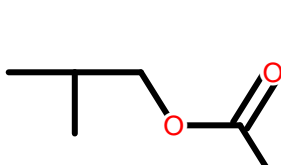

8042

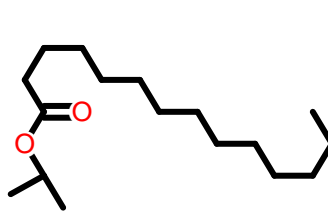

8048

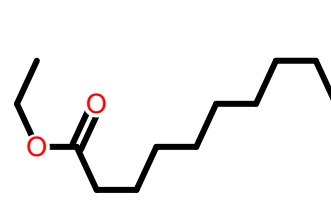

8049

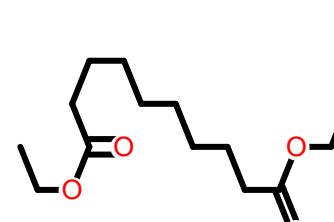

8051

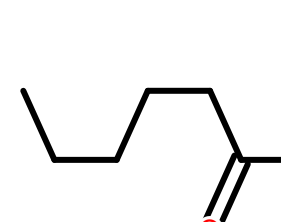

8063

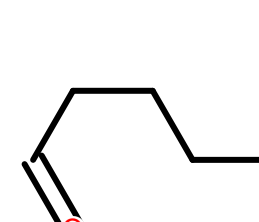

8077

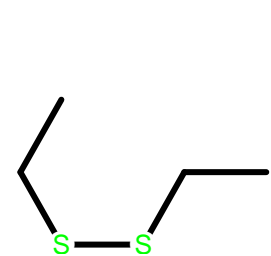

8078

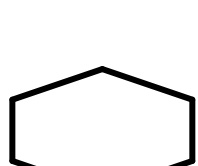

8082

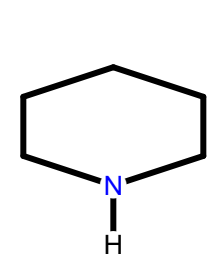

8091

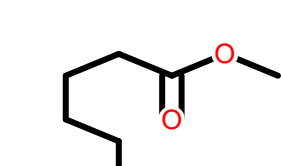

8093

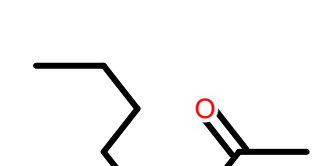

8094

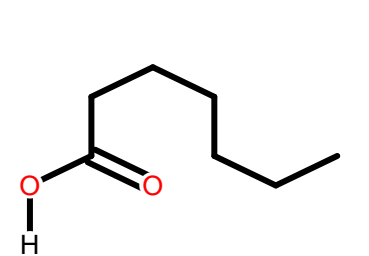

8103

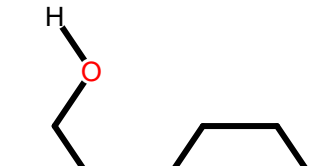

8118

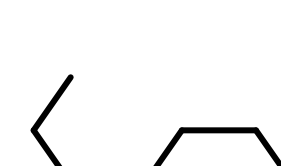

8122

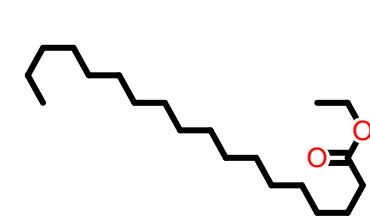

8125

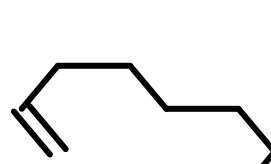

8129

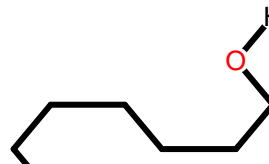

8137

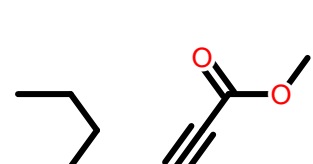

8159

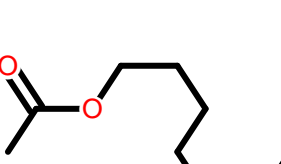

8163

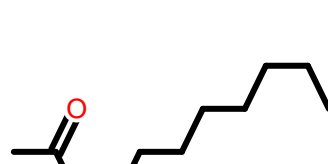

8174

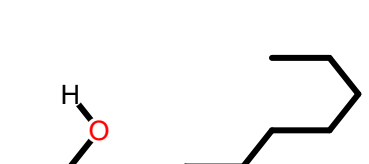

8175

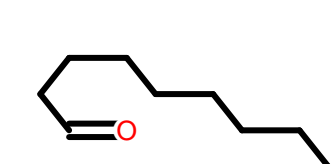

8180

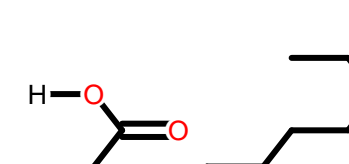

8184

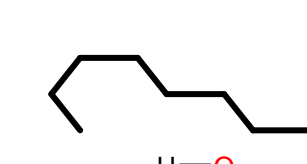

8186

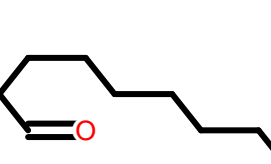

8193

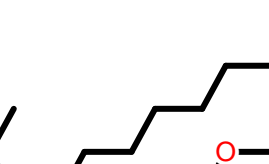

8205

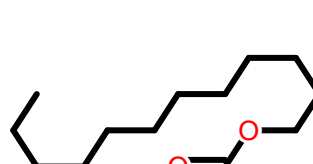

8294

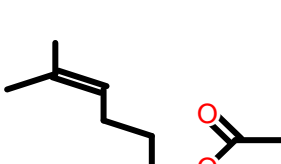

8363

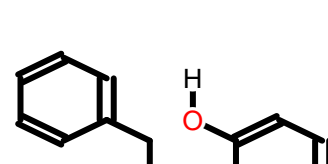

8375

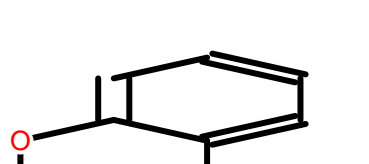

8419

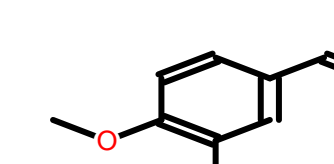

8438

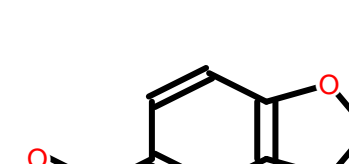

8452

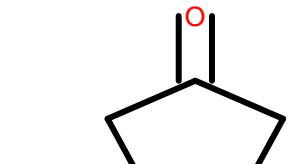

8456

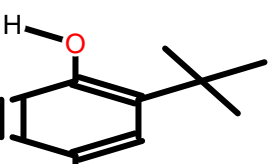

8467

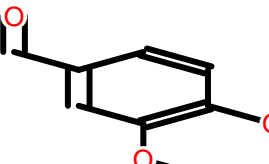

8468

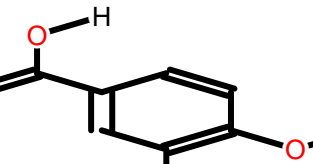

8615

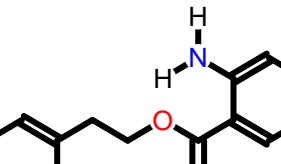

8635

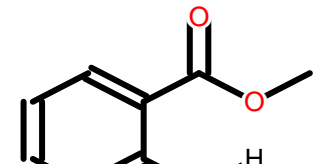

8658

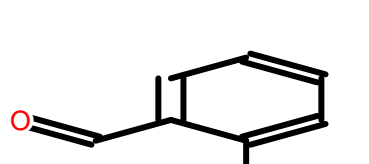

8697

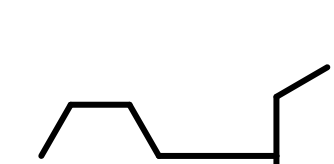

8712

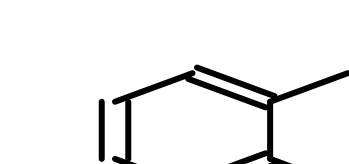

8723

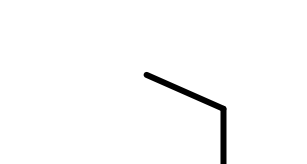

8785

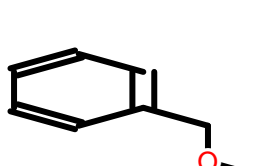

8797

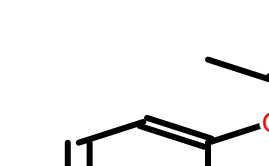

8815

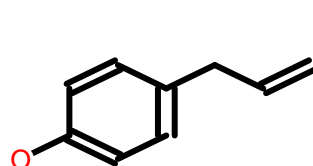

8857

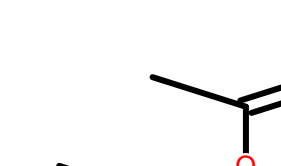

8878

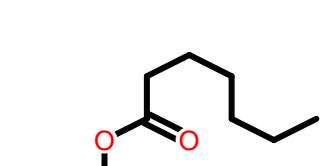

8892

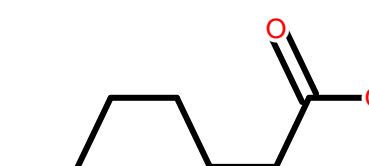

8908

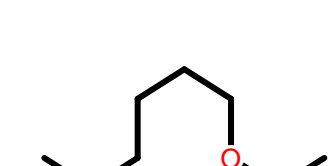

8914

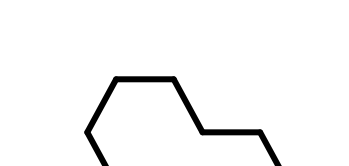

8918

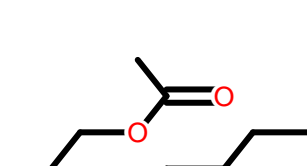

9012

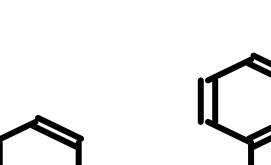

9016

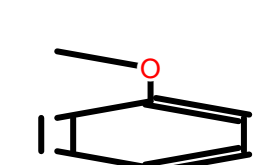

9024

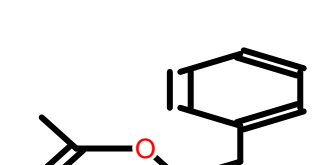

9025

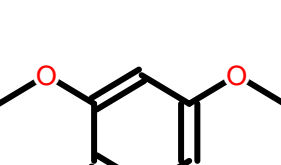

9256

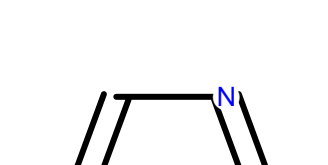

9261

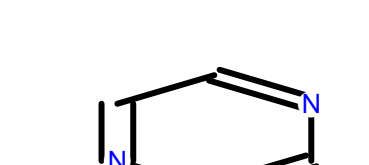

9589

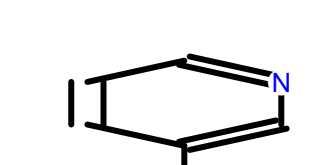

9609

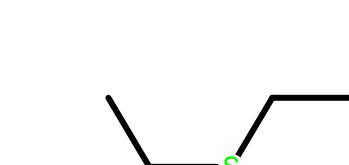

9862

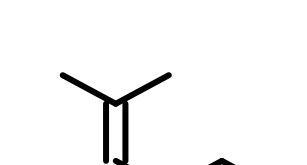

10285

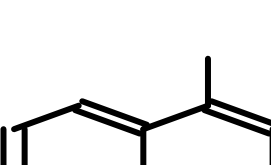

10364

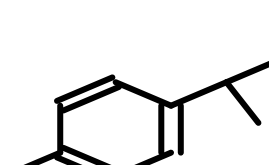

10400

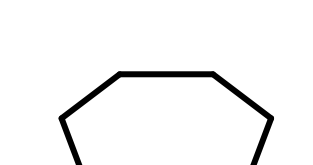

10430

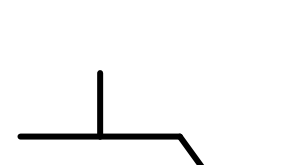

10448

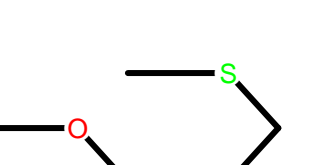

10722

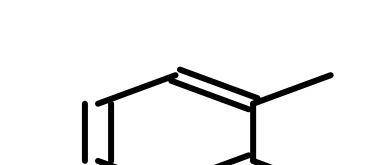

10748

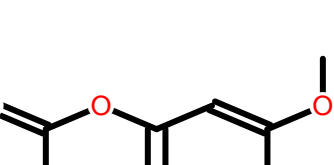

10797

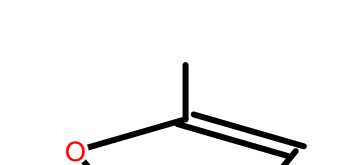

10882

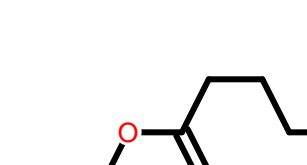

10886

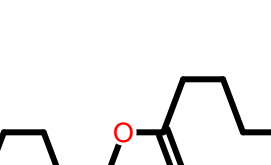

10890

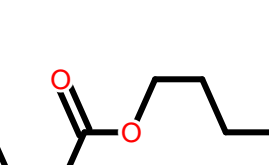

10895

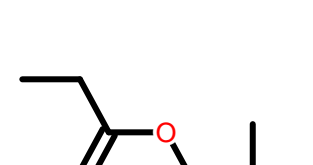

10925

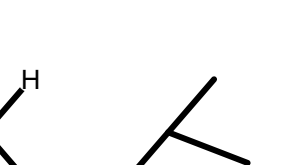

11086

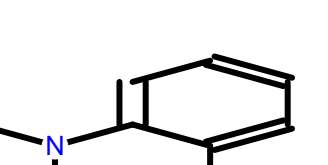

11124

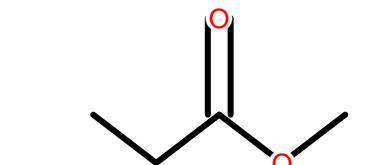

11419

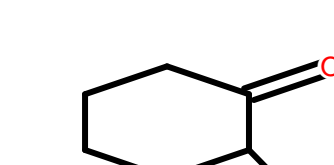

11428

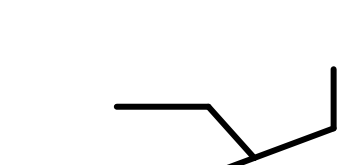

11525

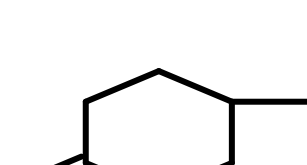

11527

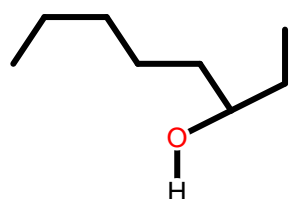

11529

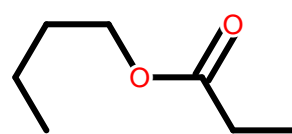

11567

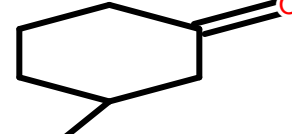

11569

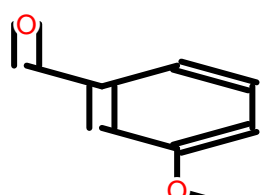

11583

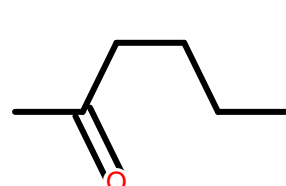

11614

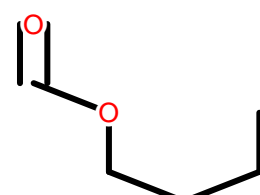

11617

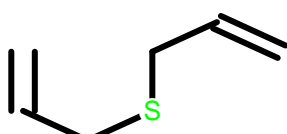

11902

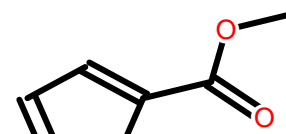

11980

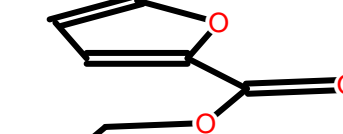

12020

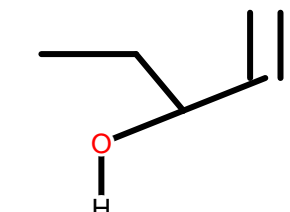

12097

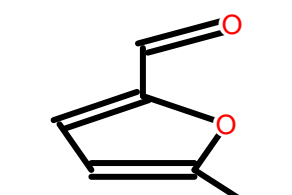

12178

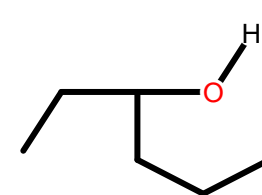

12180

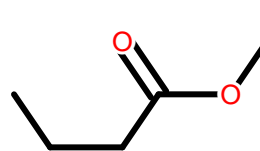

12206

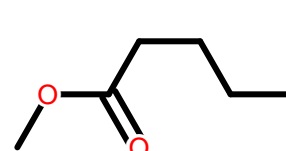

12265

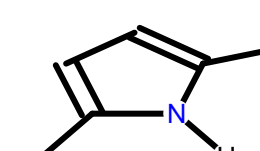

12297

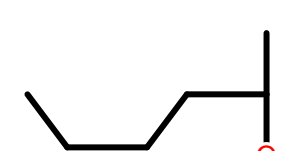

12327

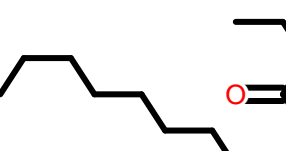

12348

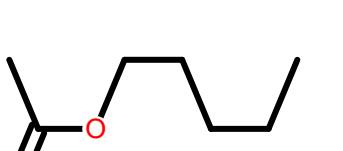

12377

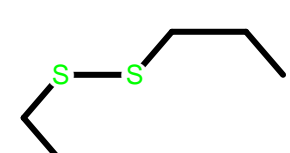

12506

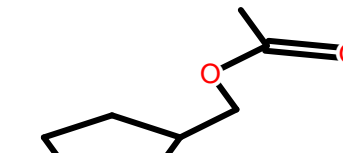

12580

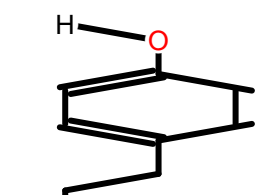

12587

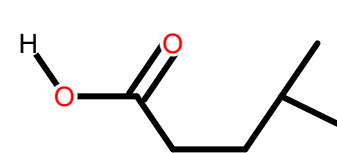

12741

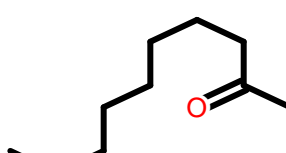

12748

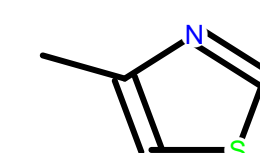

12810

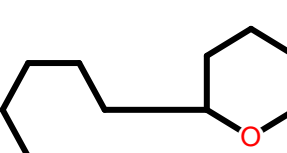

12813

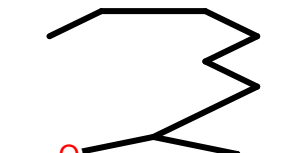

13187

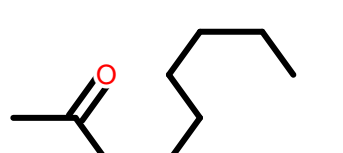

13204

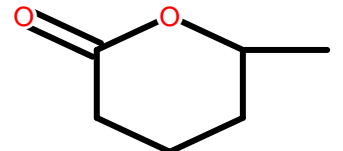

13216

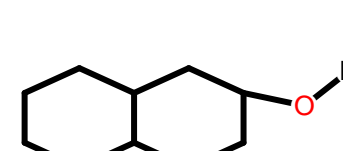

13436

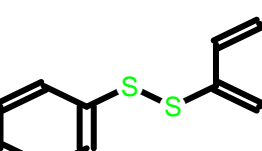

13561

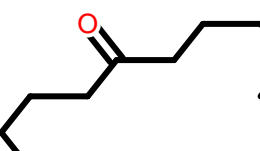

14104

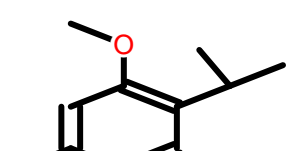

14228

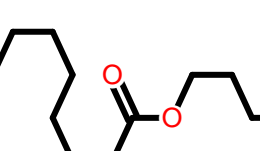

14257

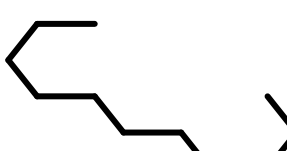

14286

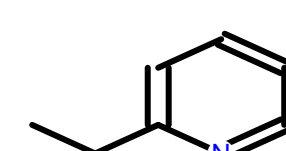

14296

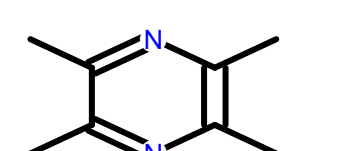

14328

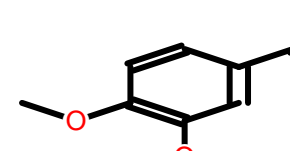

14491

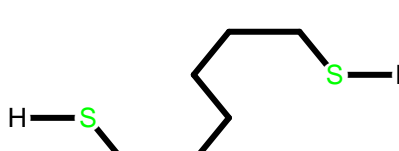

14514

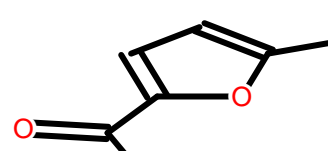

14525

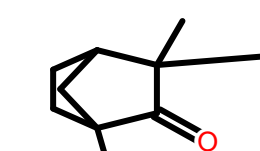

15037

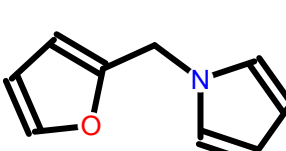

15380

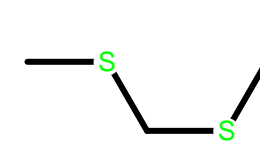

15510

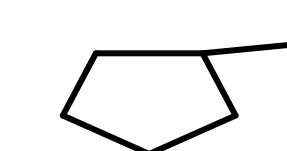

15606

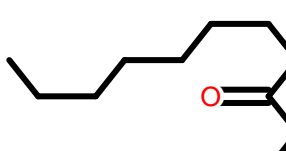

15654

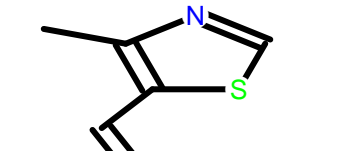

15717

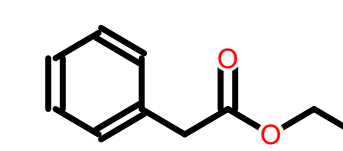

16255

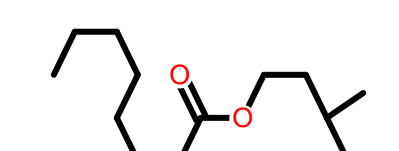

16324

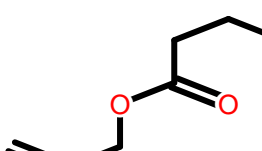

16537

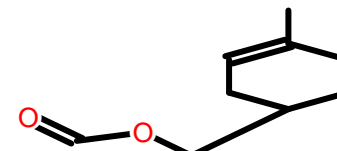

16741

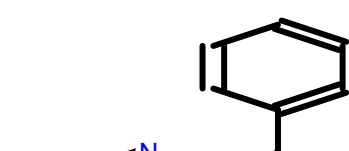

17121

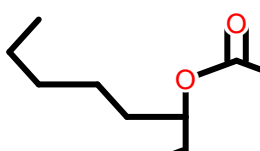

17525

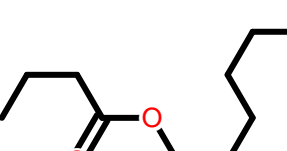

17617

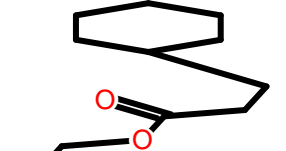

17898

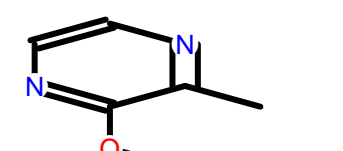

18467

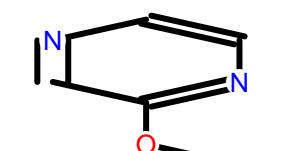

18554

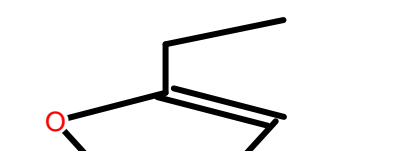

18635

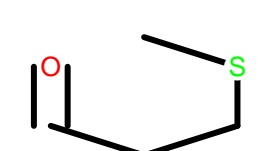

18827

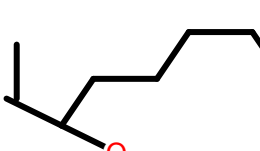

19310

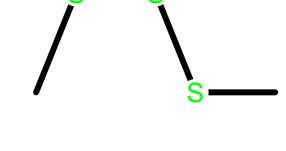

21057

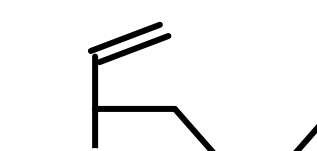

21363

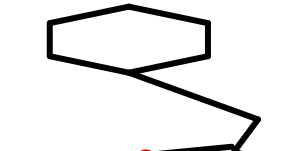

21648

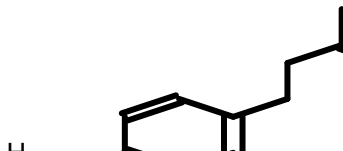

22310

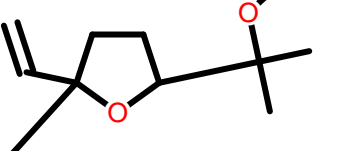

22386

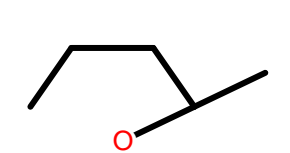

22873

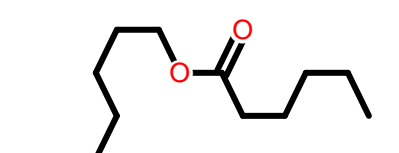

23235

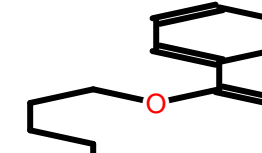

23642

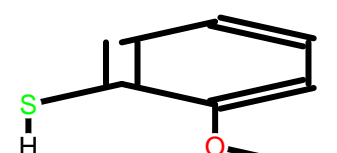

24020

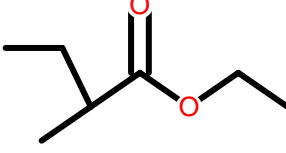

24116

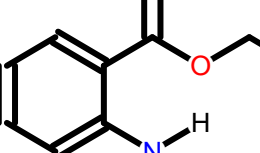

24197

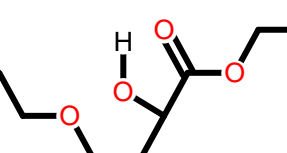

24473

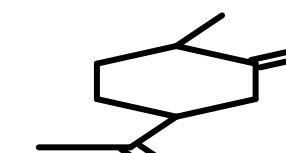

24513

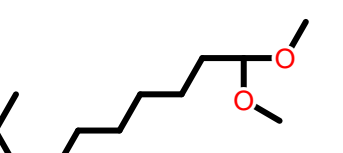

24834

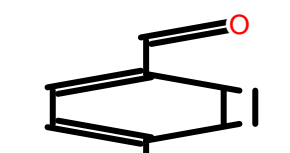

26331

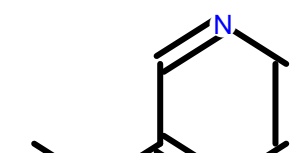

27440

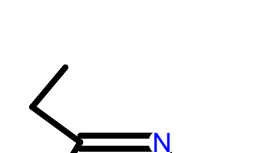

27457

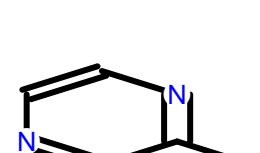

27458

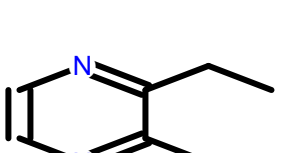

31209

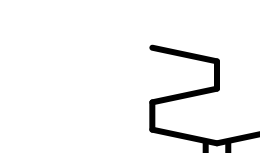

31210

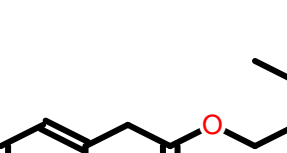

31219

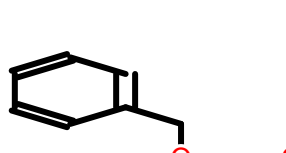

31225

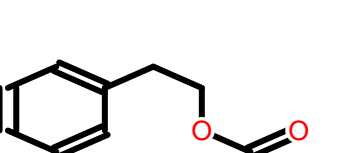

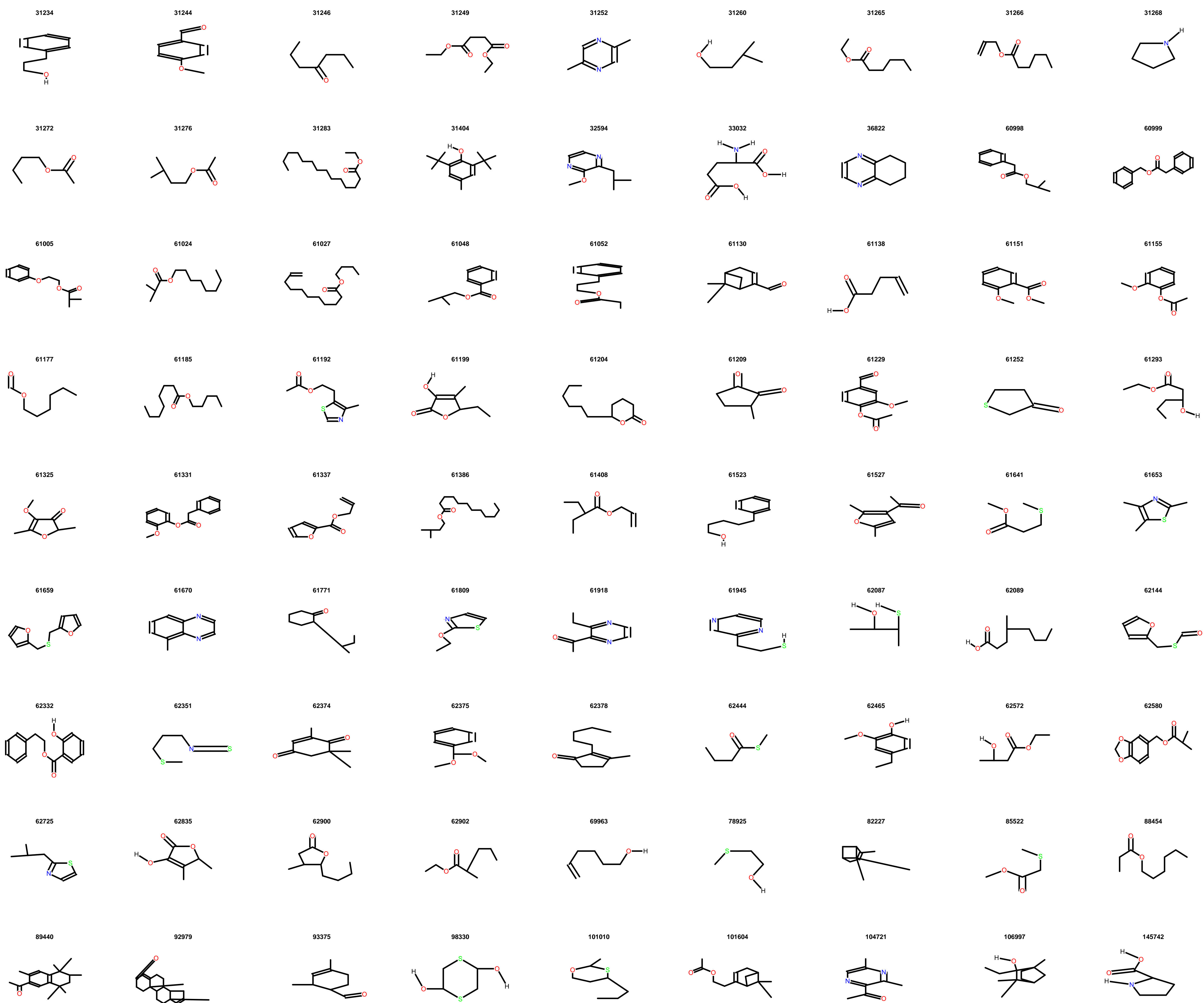

159055

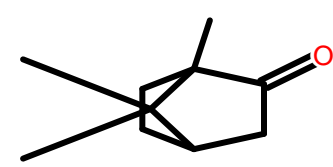

165675

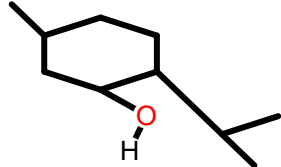

170833

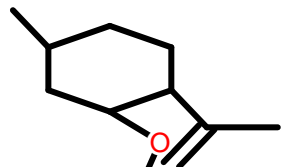

220674

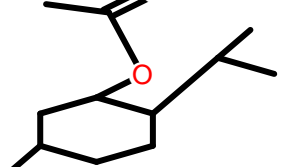

228769

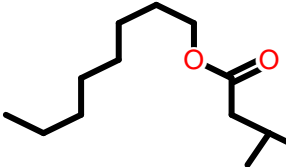

235414

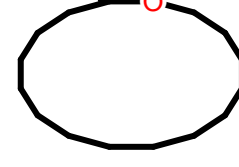

246728

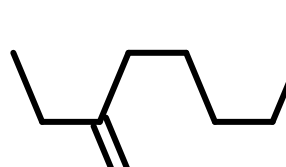

251531

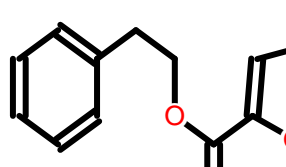

439570

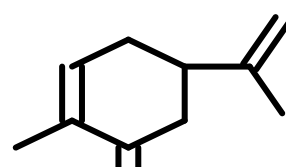

440917

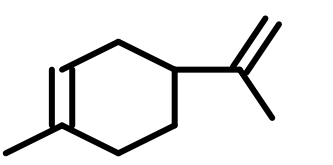

440967

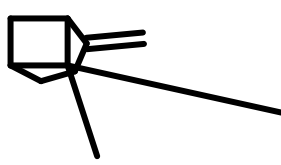

444539

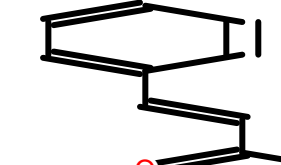

444683

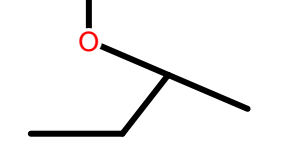

444972

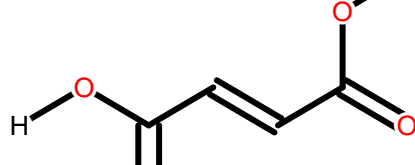

519539

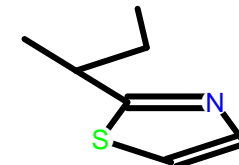

520108

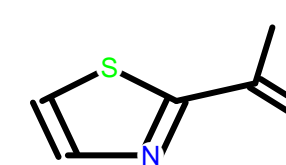

520191

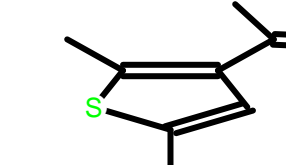

520296

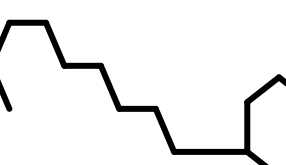

521238

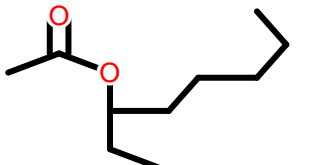

526618

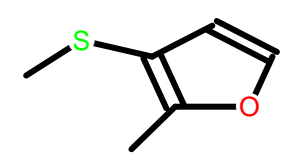

556940

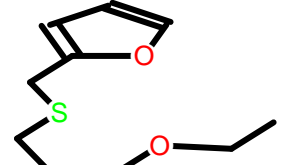

565690

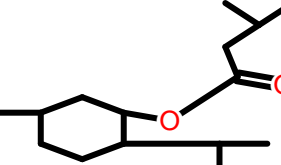

595928

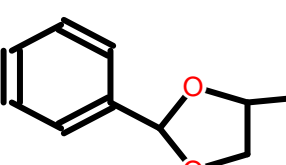

637563

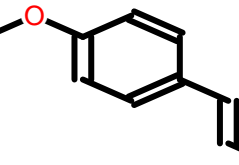

637566

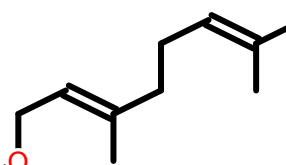

637758

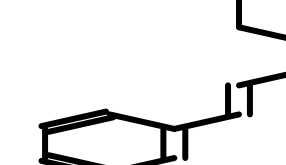

637776

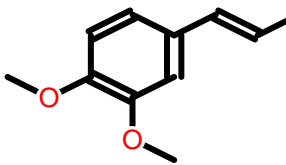

637796

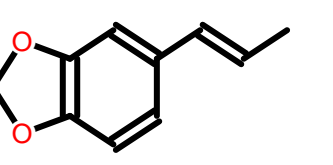

638014

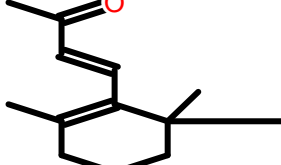

638024

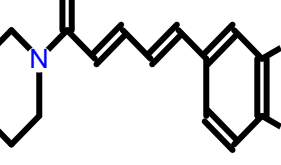

641256

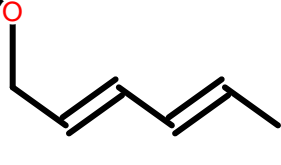

641423

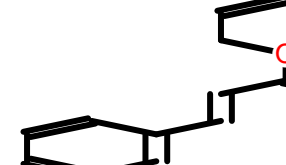

643820

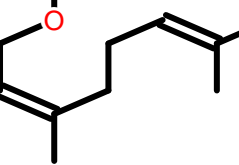

778574

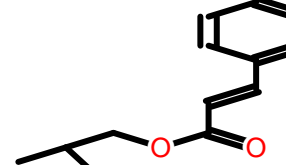

853433

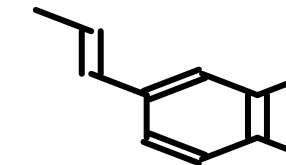

1549025

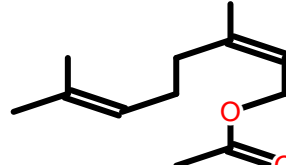

1549026

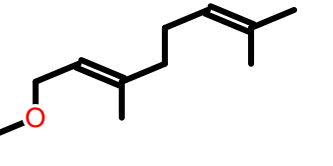

1549778

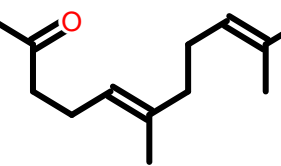

1550470

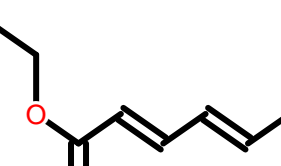

1551246

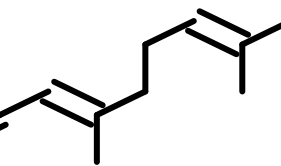

2733294

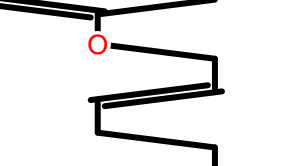

3578033

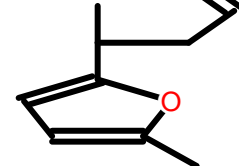

5273467

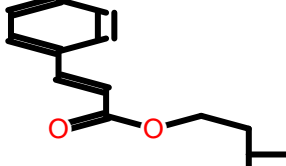

5280450

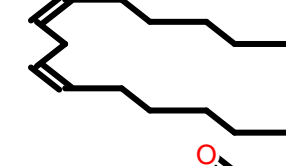

5281167

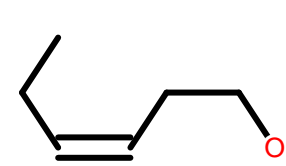

5281168

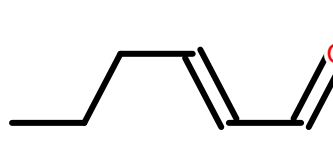

5315892

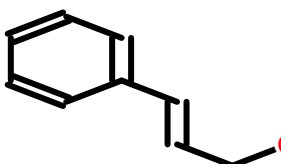

5318599

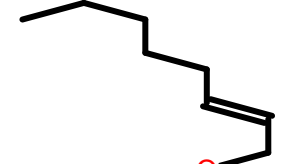

5352539

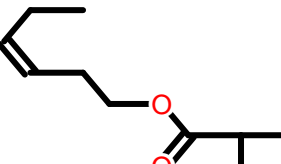

5352837

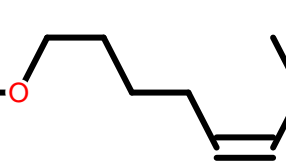

5355850

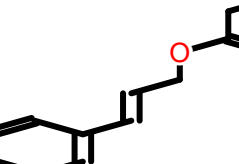

5362588

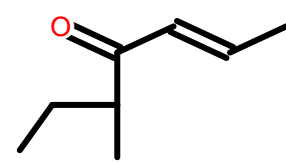

5362798

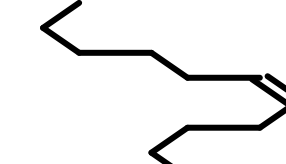

5362814

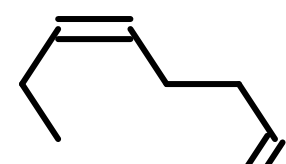

5363233

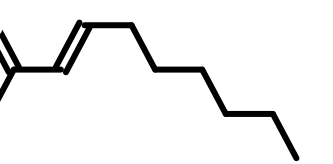

5363388

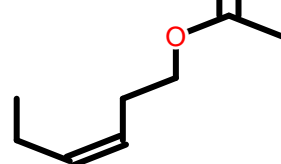

5363491

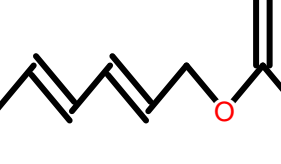

5364231

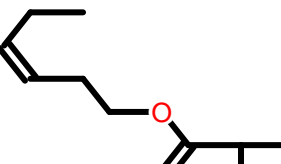

5364729

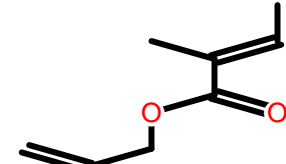

5365027

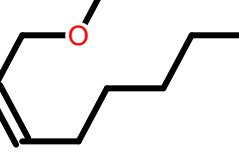

5365049

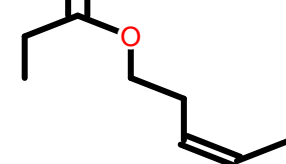

5366244

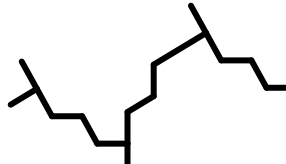

5367698

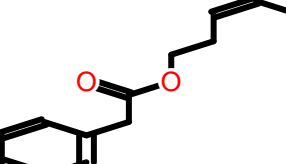

5367706

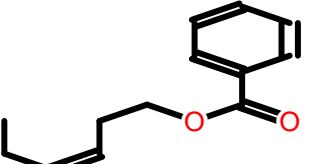

5368076

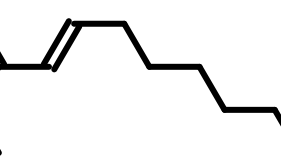

5371102

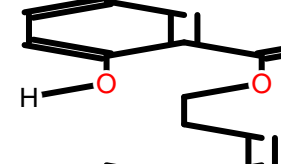

6114390

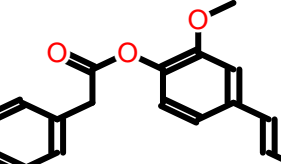

6429333

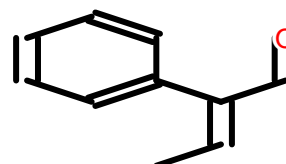

6999977

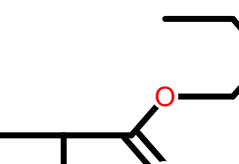

10857465

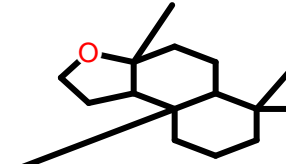

16220109

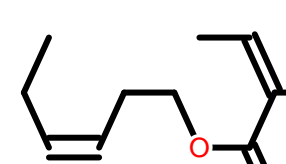

Supplement: Supplemental material [file gix127_supp.zip › Supplementary_Figure1.pdf]

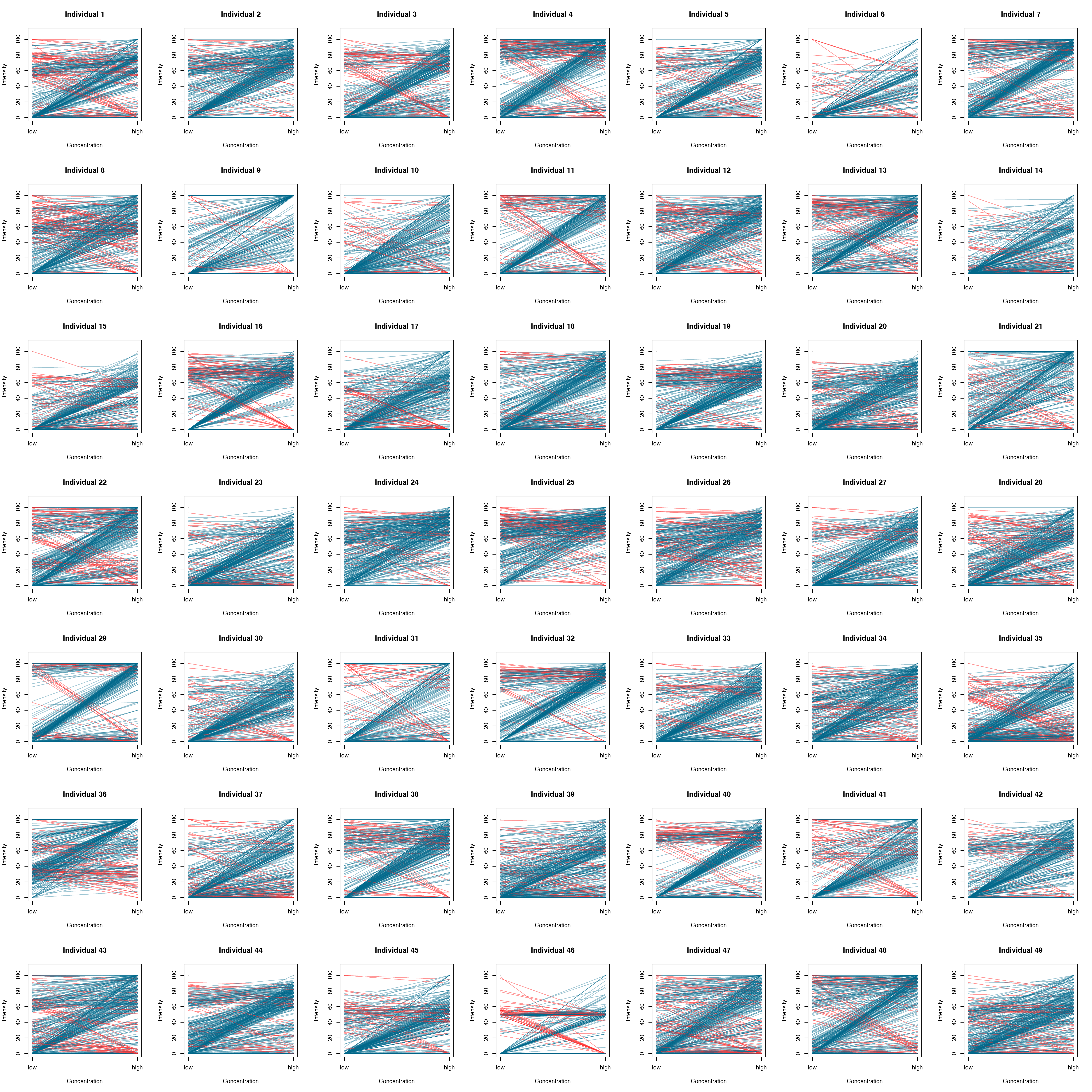

Supplement: Supplemental material [file gix127_supp.zip › Supplementary_Figure2.tif]

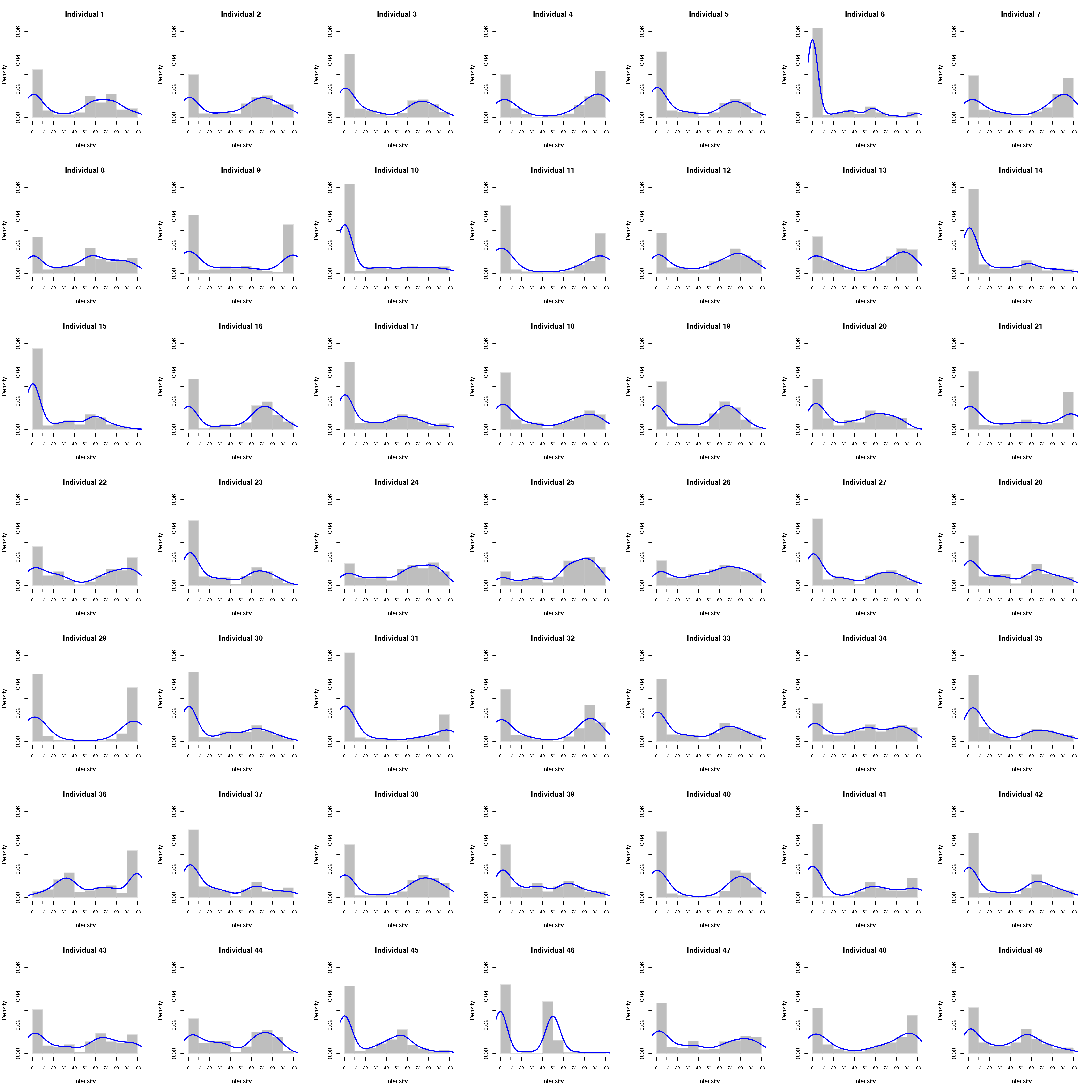

Supplement: Supplemental material [file gix127_supp.zip › Supplementary_Figure3.tif]

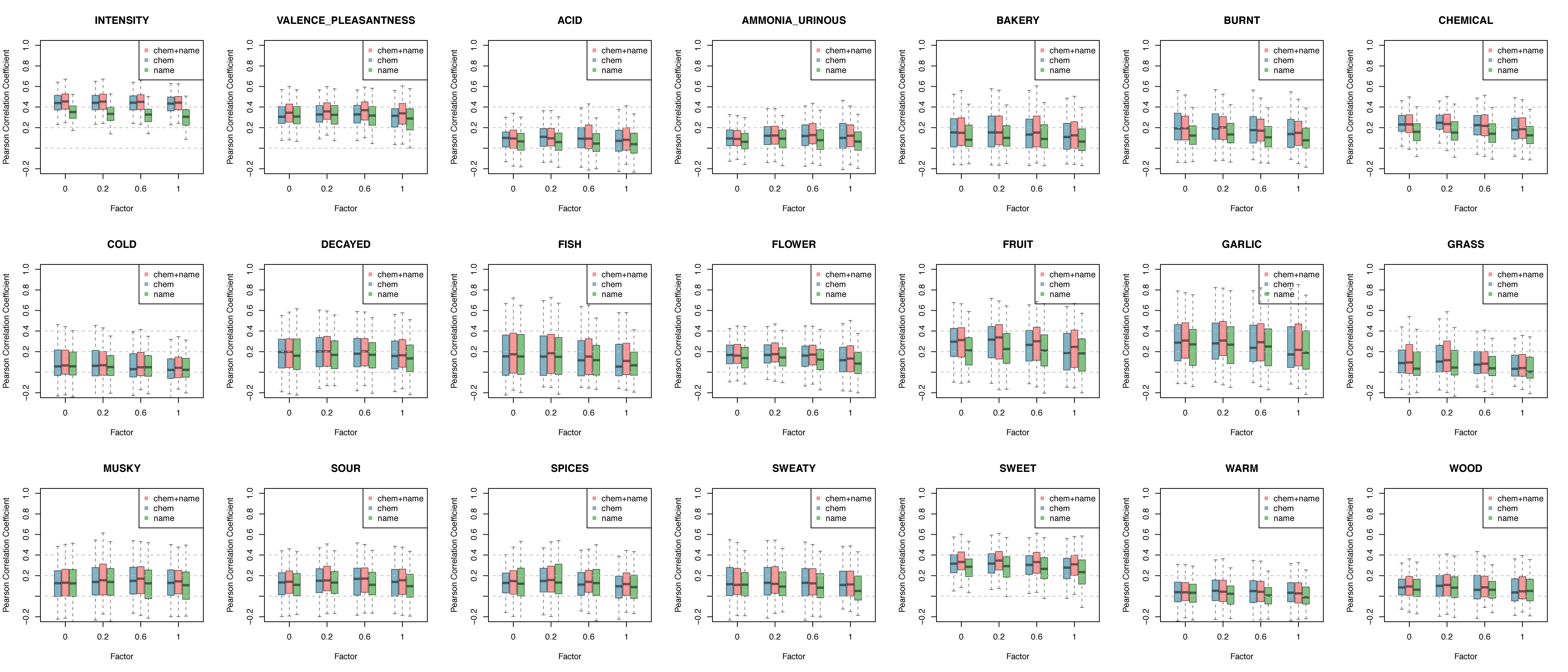

Supplement: Supplemental material [file gix127_supp.zip › Supplementary_Figure4.tif]

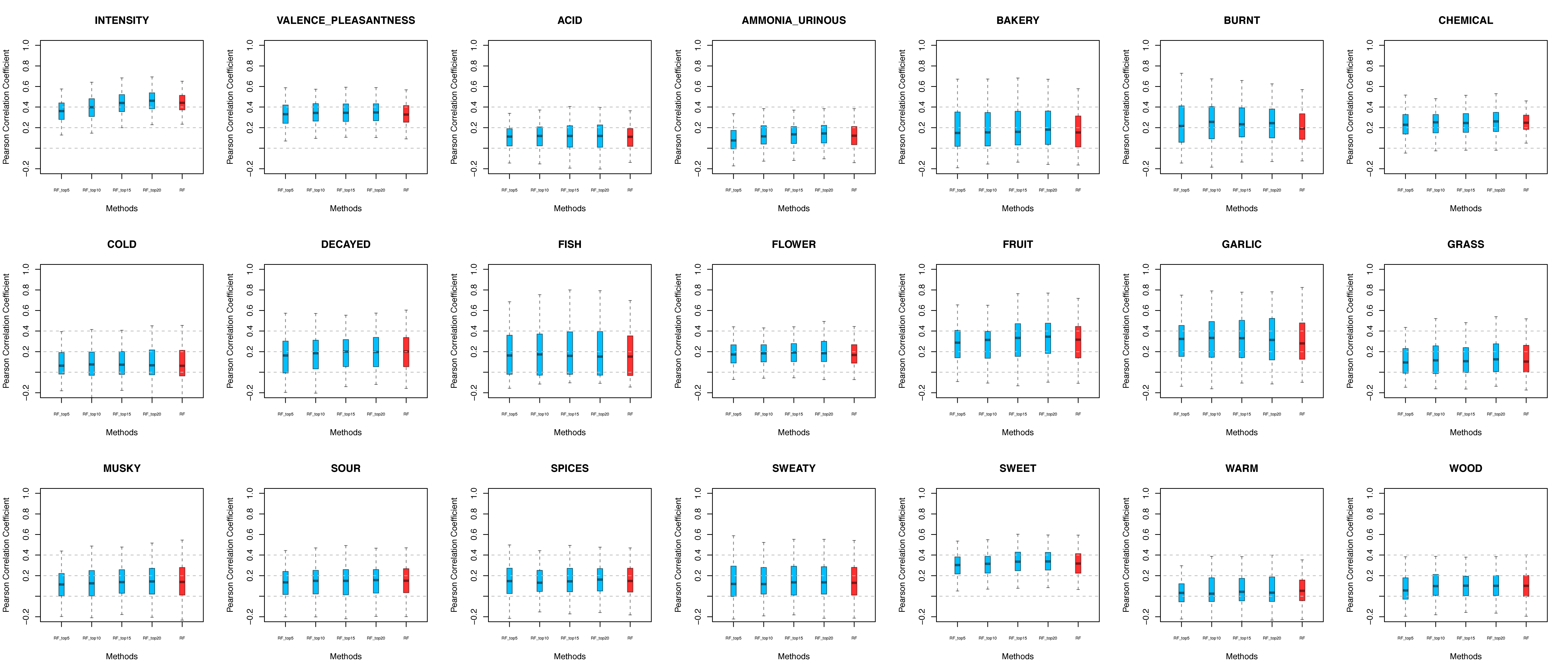

Supplement: Supplemental material [file gix127_supp.zip › Supplementary_Figure5.tif]
