# Supplementary material for: Accurate prediction of personalized olfactory perception from large-scale chemoinformatic features
Source: Gigascience. 2017 Dec 15;7(2):1–11. doi: 10.1093/gigascience/gix127 (PMC5824779; doi:10.1093/gigascience/gix127)
Supplement: Response_to_Reviewer_Comments_Original_Submission.pdf [file gix127_response_to_reviewer_comments_original_submission.pdf]

Author's Response To Reviewer Comments

Dear Editor,

Thanks for the constructive comments and suggestions from the reviewers. We've revised our manuscript "Accurate Prediction of Personalized Olfactory Perception from Large-Scale Chemoinformatic Features" accordingly, with item-by-item response below. We hope the revised manuscript significantly improved and meets the publication criteria of GigaScience.

Reviewer #2
1. Collecting and analyzing the data
The context of the study is well established; the references two previous works are adequately chosen to position the topic of the study (a reference could be added to the sentence "Different cultures have different linguistic descriptions of smell…" page 4 line 7-8).

We add references 20-22 to the sentence "Different cultures have different linguistic descriptions of smell" on page 4 line 8.

2. Data acquisition and processing
The principle of psychophysical data acquisition is well described, but there is no very precise description of the experimental protocol. At least few lines should be added in Methods part. Explanations about the supporting data are probably available at the synapse.org site, but the access seems not very simple (see below).

We add a section describing our random forest model in Method - Random forest model (page 17):

Random forest is an ensemble learning algorithm for regression and classification [32]. In a random forest, each decision tree is built from a random sampling with replacement (bootstrap samples). Furthermore, a random set of features are used to determine the best split at each node during the construction of a tree. As a result of averaging many trees (100 is used in our model), overfitting is avoided and the effects of outliers and noises are reduced.
We use perceptual ratings as targets and chemical descriptors as features to train random forest models. For the combination of 49 individuals and 21 perceptual attributes, we have 1029 (49*21) models in total. We further consider the average ratings among 49 individuals as population rating and combine it with individual rating as prediction targets to train our models (see Integrating individual and population ratings section below).

The supporting data and scripts are uploaded to Github (We can also upload them to GigaDB if necessary). The link is provided on page 22 Availability of supporting data: https://github.com/Hongyang449/olfaction_prediction_manuscript

3. Access to data and analyses tools
There is very few information concerning the 476 odorant molecules (the issue of availability of data is addressed below). This cannot be due to confidentiality of the data because of the objective of GigaScience. Nonetheless, is would it be possible to give briefly some information,

for example about the number of cyclic molecules, the number of sulfur molecules, esters, etc… Much information about molecular descriptors is available on the website of Dragon-Talete, which provides the entire list of the Dragon descriptors and their short description. The internet link www.talete.mi.it/products/dragon_molecular_descriptor_list.pdf should to be added. Because of its complex characteristics, the meaning of Dragon descriptors is not very "transparent". Nevertheless, they are fully appropriate for such studies, and the explanations of the authors are well understandable. The various examples of odorants and are clear, well chosen, and can be reproduced.

We add more descriptions about molecules to Data Description - Chemoinformatic features of molecules (page 7), including a supplementary figure of 2D structure of all molecules:
A total of 476 structurally diverse odorant molecules were used in this study, including 249 cyclic molecules, 52 sulfer molecules and 165 ester molecules (Supplementary Figure 1).

The link to the complete list of molecular descriptors is also provided at the end of this section:
The complete list of molecular descriptors is available at:
http://www.talete.mi.it/products/dragon_molecular_descriptor_list.pdf

4 & 5 Links provided for data
The internet links related to the DREAM olfaction challenge are given, but it seems that access to the dataset of molecules is not readily available (unfortunately, I was unable to find it…).
Availability of the data and source code
Several links are given, but the access seems require having a Synapse account. I begin the registration, but without any result (at least quickly). Some short precisions could be added in Availability of supporting data, or in Methods part to allow direct access to data and code source.

The supporting data and scripts are uploaded to Github (We can also upload them to GigaDB if necessary). The link is provided on page 22 Availability of supporting data:
https://github.com/Hongyang449/olfaction_prediction_manuscript

6. Quality of the data
I don't have another remarks than those made above.

7. Analysis and discussion of the results
The analysis of the results and their explanation are clearly stated and well discussed. I did not noticed positively or negatively biased interpretations.
I just have a reservation with regard to the sentence "These structural analogs with different odors are clearly separated in 1 the 2-dimensional feature spaces" (legend of Fig. 6 page 22 lines 1-2). I globally agree, but some separations are not so clear (Fig. 6). For example, the "decayed" character of furoate ester 2 (Figure 6A) differs from the furoate 1 and 3 (radar chart middle panel); nevertheless the three points are close on 2D projection (right panel). Conversely, the amino-acids 4 and 6 show similar "sour" quality with regard to radar chart, and strongly differ from leucine 5, but the three points are separated on 2D projection. That also reveals the relative limit of the 2D projections. In any case, this sentence would to be qualified, with the risk to add complexity, all the more so as this is in the figure legend. I would be inclined to delete to avoid unnecessary confusion and wrong perceptions.

We delete the last sentence in the legend of Figure 6.

8. Appropriateness of methods

The comparison among the methods is well explained, the choice of non-linear methods, and especially random forest, seems judicious. However, I known the principle, but I am myself not familiar with the experimental use of such method. Thus I have some questions. For example, might possibly be provided several other statistical parameters in addition to DeltaError and Pearson coefficient? Could they available at the synapse.org web site? I identified 281 molecular descriptors on the basis of Supplemental Tables 2 and 3: would it be possible to provide their values (in a supplemental table)? It would therefore be beneficial to provide some short additional explanation concerning the random forest method.

We add a Supplementary Table 4, which provides both Pearson's and Spearman's correlations of top 500 features ranked by Pearson's correlation.
We add a section describing our random forest model in Method - Random forest model (page 17).

9. Strengths and weaknesses of the methods

It seems that not additional experiments are needed.

10. Practices in reporting standards

As discussed above, the authors provide internet links that aim to provide access to data and code source of used algorithm. Nevertheless, this access seems a little problematic for non-participants to DREAM Olfaction Prediction Challenge and/or not familiar with synapse.org.

11. Presentation and organization of the manuscript

Other than my previous remarks concerning deliverable list of molecules and related descriptors values, there is no issue concerning the presentation and organization of the manuscript. The manuscript is clear and reads well. The figures are clear. Their analysis and understanding requires sometimes an effort, which is fully justified by the information provided (especially Figure 6).

12. Requested revisions

The requested revisions relate mainly to the means to facilitate the availability of the data, by either specific internet links providing easily the information or by additional tables. Some other minor changes are suggested in the comments above.

13. Ethical question

There is no description of the sensory data acquisition. The rules and laws concerning the human participants vary from country to country; a few words about ethical conditions of the sensory study could be added.

According to the original study (reference 33), we add the following sentence to page 6 line 6-7: These subjects volunteered and gave their written informed consent to smell the stimuli used in this study [33].

Reviewer #3

1. At Pg6 The explanation of descriptors of the study is unclear see Psychophysical dataset section. 49 participants/subjects were asked to rate 476 different molecules that were provided at two different concentrations. Out of 476 molecules, 20 molecules were rated or tested twice. In total there are 992 molecules, where each molecule is present at two different concentrations.
a) Authors did not suggest the different molecular concentrations in this study. Is it necessary to combine the two different concentration together? It is not clear form the manuscript that what is the benefit different concentrations brings to the model?

We add the following sentence to page 17 line 20-22:
Since the original number of sample (407) is relatively small, combining high and low concentrations doubles the sample size and this step is crucial to achieve high performance.

b) The 20 replicated molecules may add biased in your model prediction. Do you find a major difference between the replicated ratings for 20 molecules? Why were these 20 molecules selected?

We add the following sentences to page 17 line 22-23 and page 18 line 1-2:
There are 20 replicated molecules, which were selected in the original Rockefeller University Smell Study. In general, the ratings were consistent between two replicates [33]. In our study, we treat replicates as separate examples since the fraction is small (20/407=0.049), which doesn't affect the results.

c) At line 18-20, the author discusses the training set, leaderboard and test set. It is unclear the involvement of individual ratings in the training set. As I understand that each participant had to rate every molecule in respect to all the semantic attributes between 0-100. The question is how the individual ratings are included in the model prediction is unclear. Whether the scaling was performed individually for participants before average the value as a second step?

We add a section describing our model in Method - Random forest model (page 17). We didn't scale individual ratings.

2. There is no explanation provided for delta error? Usually, in random forest to calculate the variable importance the mean square error on the out-of-bag (OOB) for each tree or Mean Decrease Accuracy or Mean Decrease Gini for each tree is calculated. This will help to judge the performance.

We explain delta error in page 11 line 1-3:
Random forest enables us to estimate the importance of each chemical feature by permuting the values of a feature across samples and computing the increase in prediction error. We calculate the increased delta error of each chemical feature for all 21 olfactory qualities.

3. Pg 12 .14 = "different number of key features" - this phrase is vague clarification is needed.

We modify this sentence and provide exact numbers:
Rebuilding the random forest model with top 5,10,15 or 20 key features, we find that a small set of chemical features is sufficient for accurate prediction.

4. Pg10.9 Author says "To further improve the performance, we applied a sliding window of 4-letter size to each molecule name, generating a total of 11,786 binary name features". On the contrary, we see that the results are not improved. I fail to understand why this analysis was performed. Moreover, why the 4-letter window was selected, one can test different window size. Still, i don't see an immediate improvement in the final model.

We add a Supplementary Figure 5 to demonstrate the improved performances using name features. For intensity, valence and many somatic attributes (e.g. fruit, galic, sweet), the name features improve the final results.
To explain 4-letter window, we add the following sentences to page 10 line 10-14:
These name features are very similar to the molecular fingerprints, providing extra information about the similarities of molecules. The 4-letter window is selected to efficiently capture the chemical similarity. Larger sliding window sizes greatly increase computational cost, since the size of feature space is an exponential function of window size.

5. It is not written which scripting language the algorithm was designed.

Our scripts are written in perl, R, python and matlab.
The supporting data and scripts are uploaded to Github (We can also upload them to GigaDB if necessary). The link is provided on page 22 Availability of supporting data:
https://github.com/Hongyang449/olfaction_prediction_manuscript

6. Method section
a) Check the equation at line pg 17.12
b) Check the equation at pg 18.6

The equations in the word format manuscript are shown correctly. We think this is caused by format conversion from word to pdf.

c) The parameter selection step is missing and/or is not explained in the paper. In your analysis you are comparing different learning algorithm, the different parameters should be reported. This will help the reader to understand the performance.

We add the parameters used in different models in Method - Selection of base learner (page 18):
The regularization alpha in ridge is 10. The penalty parameter C and coefficient gamma of the SVM rbf kernel are 1000 and 0.01, respectively. All other parameters are the default ones.
