# Supplementary material for: Accurate prediction of personalized olfactory perception from large-scale chemoinformatic features
Source: Gigascience. 2017 Dec 15;7(2):1–11. doi: 10.1093/gigascience/gix127 (PMC5824779; doi:10.1093/gigascience/gix127)
Supplement: Reviewer_2_Report_(Original_Submission) -- Anne Tromelin — 30 Jun 2017 Reviewed [file gix127_reviewer_2_report_(original_submission).pdf]

Reviewer Report

Title: Accurate Prediction of Personalized Olfactory Perception from Large-Scale Chemoinformatic Features

Version: Original Submission **Date:** 6/30/2017

Reviewer name: Anne Tromelin

Reviewer Comments to Author:

1. Collecting and analyzing the data
The context of the study is well established; the references two previous works are adequately chosen to position the topic of the study (a reference could be added to the sentence "Different cultures have different linguistic descriptions of smell…" page 4 line 7-8).
In the opinion of the authors themselves, a set of 476 odorant molecules corresponds to a relatively small-sized training set. There are several larger odorants bases, as the Arctander database, but at the opposite a very large disease chemical databases, which encompass tens or even hundreds of thousands of molecules, the number of odorant molecules described odorant databases do not exceed a few thousand molecules (this could be around 10%, 1% or even below than the total, and unknown, number of possible odorants). Consequently, the present training set represents about 10% of known odorants. Nevertheless, the reported data are based on the assessments given by 49 subjects, which represent about 1000 stimuli. Recording data for more than 1000 or 2000 odorants involving more than 50 subjects would be difficult, if not impossible, to achieve within reasonable time. Broadly speaking, there are two options. Either use a medium-sized training set of odorants assessed by several dozen of subjects, which provides precise data, or 5-10 times larger set of odorants whose description results from a compilation of results obtained by several sensory analyses involving various groups of subjects. In my view, the two options have advantages and limitations, and a balance between these two types of studies (small detailed dataset vs large database) in the literature is a good compromise. In this context, the use of a relatively small size dataset is fully justified.
The design of any molecule with a specific target odor will still requires much works and studies. In that challenge, the presented results are very interesting and give promising perspectives. In my view, in addition to the great interest of the results, there are two key points mentioned in the manuscript. The first relates to the remarkable variability of olfactory perception among individuals (very clearly illustrated), and the strategy of the authors to overcome it. The second raises the issue of the nature of molecular descriptors, and rightly emphases the little power of simple chemical features, which refer to a too rigid -and probably obsolete- understanding of the lock-and-key model.

2. Data acquisition and processing
The principle of psychophysical data acquisition is well described, but there is no very precise description of the experimental protocol. At least few lines should be added in Methods part. Explanations about the supporting data are probably available at the synapse.org site, but the access seems not very simple (see below).

3. Access to data and analyses tools

There is very few information concerning the 476 odorant molecules (the issue of availability of data is addressed below). This cannot be due to confidentiality of the data because of the objective of GigaScience. Nonetheless, is would it be possible to give briefly some information, for example about the number of cyclic molecules, the number of sulfur molecules, esters, etc…

Much information about molecular descriptors is available on the website of Dragon-Talete, which provides the entire list of the Dragon descriptors and their short description. The internet link www.talete.mi.it/products/dragon_molecular_descriptor_list.pdf should to be added. Because of its complex characteristics, the meaning of Dragon descriptors is not very "transparent". Nevertheless, they are fully appropriate for such studies, and the explanations of the authors are well understandable. The various examples of odorants and are clear, well chosen, and can be reproduced.

4. Links provided for data

The internet links related to the DREAM olfaction challenge are given, but it seems that access to the dataset of molecules is not readily available (unfortunately, I was unable to find it…).

5. Availability of the data and source code

Several links are given, but the access seems require having a Synapse account. I begin the registration, but without any result (at least quickly). Some short precisions could be added in Availability of supporting data, or in Methods part to allow direct access to data and code source.

6. Quality of the data

I don't have another remarks than those made above.

7. Analysis and discussion of the results

The analysis of the results and their explanation are clearly stated and well discussed. I did not noticed positively or negatively biased interpretations.

I just have a reservation with regard to the sentence "These structural analogs with different odors are clearly separated in 1 the 2-dimensional feature spaces" (legend of Fig. 6 page 22 lines 1-2). I globally agree, but some separations are not so clear (Fig. 6). For example, the "decayed" character of furoate ester 2 (Figure 6A) differs from the furoate 1 and 3 (radar chart middle panel); nevertheless the three points are close on 2D projection (right panel). Conversely, the amino-acids 4 and 6 show similar "sour" quality with regard to radar chart, and strongly differ from leucine 5, but the three points are separated on 2D projection. That also reveals the relative limit of the 2D projections. In any case, this sentence would to be qualified, with the risk to add complexity, all the more so as this is in the figure legend. I would be inclined to delete to avoid unnecessary confusion and wrong perceptions.

8. Appropriateness of methods

The comparison among the methods is well explained, the choice of non-linear methods, and especially random forest, seems judicious. However, I known the principle, but I am myself not familiar with the experimental use of such method. Thus I have some questions. For example, might possibly be provided

several other statistical parameters in addition to DeltaError and Pearson coefficient? Could they available at the synapse.org web site? I identified 281 molecular descriptors on the basis of Supplemental Tables 2 and 3: would it be possible to provide their values (in a supplemental table)? It would therefore be beneficial to provide some short additional explanation concerning the random forest method.

9. Strengths and weaknesses of the methods
It seems that not additional experiments are needed.

10. Practices in reporting standards
As discussed above, the authors provide internet links that aim to provide access to data and code source of used algorithm. Nevertheless, this access seems a little problematic for non-participants to DREAM Olfaction Prediction Challenge and/or not familiar with synapse.org.

11. Presentation and organization of the manuscript
Other than my previous remarks concerning deliverable list of molecules and related descriptors values, there is no issue concerning the presentation and organization of the manuscript. The manuscript is clear and reads well. The figures are clear. Their analysis and understanding requires sometimes an effort, which is fully justified by the information provided (especially Figure 6).

12. Requested revisions
The requested revisions relates mainly to the means to facilitate the availability of the data, by either specific internet links providing easily the information or by additional tables. Some other minor changes are suggested in the comments above.

13. Ethical question
There is no description of the sensory data acquisition. The rules and laws concerning the human participants vary from country to country; a few words about ethical conditions of the sensory study could be added.

Methods

Are the methods appropriate to the aims of the study, are they well described, and are necessary controls included? Yes

Conclusions

Are the conclusions adequately supported by the data shown? Yes

Reporting Standards

Does the manuscript adhere to the journal's guidelines on [minimum standards of reporting?] NoChoose an item.

Statistics

Are you able to assess all statistics in the manuscript, including the appropriateness of statistical tests used? No, and I do not feel adequately qualified to assess the statistics.

Quality of Written English

Please indicate the quality of language in the manuscript: Acceptable

Declaration of Competing Interests

Please complete a declaration of competing interests, considering the following questions:

- Have you in the past five years received reimbursements, fees, funding, or salary from an organisation that may in any way gain or lose financially from the publication of this manuscript, either now or in the future?
- Do you hold any stocks or shares in an organisation that may in any way gain or lose financially from the publication of this manuscript, either now or in the future?
- Do you hold or are you currently applying for any patents relating to the content of the manuscript?
- Have you received reimbursements, fees, funding, or salary from an organization that holds or has applied for patents relating to the content of the manuscript?
- Do you have any other financial competing interests?
- Do you have any non-financial competing interests in relation to this paper?

If you can answer no to all of the above, write 'I declare that I have no competing interests' below. If your reply is yes to any, please give details below.

declare that I have no competing interests

I agree to the open peer review policy of the journal. I understand that my name will be included on my report to the authors and, if the manuscript is accepted for publication, my named report including any attachments I upload will be posted on the website along with the authors' responses. I agree for my report to be made available under an Open Access Creative Commons CC-BY license (http://creativecommons.org/licenses/by/4.0/). I understand that any comments which I do not wish to be included in my named report can be included as confidential comments to the editors, which will not be published.

I agree to the open peer review policy of the journal

To further support our reviewers, we have joined with Publons, where you can gain additional credit to further highlight your hard work (see: https://publons.com/journal/530/gigascience). On publication of

this paper, your review will be automatically added to Publons, you can then choose whether or not to claim your Publons credit. I understand this statement.

Yes
