# Supplementary material for: Accurate prediction of personalized olfactory perception from large-scale chemoinformatic features
Source: Gigascience. 2017 Dec 15;7(2):1–11. doi: 10.1093/gigascience/gix127 (PMC5824779; doi:10.1093/gigascience/gix127)
Supplement: Reviewer_3_Report_(Original_Submission) -- Neetika Nath — 10 Aug 2017 Reviewed [file gix127_reviewer_3_report_(original_submission).pdf]

Reviewer Report

Title: Accurate Prediction of Personalized Olfactory Perception from Large-Scale Chemoinformatic Features

Version: Original Submission **Date:** 8/10/2017

Reviewer name: Neetika Nath

Reviewer Comments to Author:

The authors of the paper " Accurate prediction of personalized olfactory perception from large-scale chemoinformatic features" studied various odorants to predict individual observed ratings using random forest model. In addition to a large amount of the dataset, the authors have compared the performance of four different learning algorithm and realized that random forest outperforms others. However, the publication lacks the description of the random forest model.

Comments:

1. At Pg6 The explanation of descriptors of the study is unclear see Psychophysical dataset section. 49 participants/subjects were asked to rate 476 different molecules that were provided at two different concentrations. Out of 476 molecules, 20 molecules were rated or tested twice. In total there are 992 molecules, where each molecule is present at two different concentrations.
a) Authors did not suggest the different molecular concentrations in this study. Is it necessary to combine the two different concentration together? It is not clear form the manuscript that what is the benefit different concentrations brings to the model?
b) The 20 replicated molecules may add biased in your model prediction. Do you find a major difference between the replicated ratings for 20 molecules? Why were these 20 molecules selected?
c) At line 18-20, the author discusses the training set, leaderboard and test set. It is unclear the involvement of individual ratings in the training set. As I understand that each participant had to rate every molecule in respect to all the semantic attributes between 0-100. The question is how the individual ratings are included in the model prediction is unclear. Whether the scaling was performed individually for participants before average the value as a second step?

2. There is no explanation provided for delta error? Usually, in random forest to calculate the variable importance the mean square error on the out-of-bag (OOB) for each tree or Mean Decrease Accuracy or Mean Decrease Gini for each tree is calculated. This will help to judge the performance.

3. Pg 12 .14 = "different number of key features" - this phrase is vague clarification is needed.

4. Pg10.9 Author says "To further improve the performance, we applied a sliding window of 4-letter size to each molecule name, generating a total of 11,786 binary name features". On the contrary, we see that the results are not improved. I fail to understand why this analysis was performed. Moreover, why

the 4-letter window was selected, one can test different window size. Still, i don't see an immediate improvement in the final model.

5. It is not written which scripting language the algorithm was designed.

6. Method section
a) Check the equation at line pg 17.12
b) Check the equation at pg 18.6
c) The parameter selection step is missing and/or is not explained in the paper. In your analysis you are comparing different learning algorithm, the different parameters should be reported. This will help the reader to understand the performance.

Methods

Are the methods appropriate to the aims of the study, are they well described, and are necessary controls included? No

Conclusions

Are the conclusions adequately supported by the data shown? Yes

Reporting Standards

Does the manuscript adhere to the journal's guidelines on minimum standards of reporting? NoChoose an item.

Statistics

Are you able to assess all statistics in the manuscript, including the appropriateness of statistical tests used? Yes, and I have assessed the statistics in my report.

Quality of Written English

Please indicate the quality of language in the manuscript: Acceptable

Declaration of Competing Interests

Please complete a declaration of competing interests, considering the following questions:

- Have you in the past five years received reimbursements, fees, funding, or salary from an organisation that may in any way gain or lose financially from the publication of this manuscript, either now or in the future?
- Do you hold any stocks or shares in an organisation that may in any way gain or lose financially from the publication of this manuscript, either now or in the future?

- Do you hold or are you currently applying for any patents relating to the content of the manuscript?
- Have you received reimbursements, fees, funding, or salary from an organization that holds or has applied for patents relating to the content of the manuscript?
- Do you have any other financial competing interests?
- Do you have any non-financial competing interests in relation to this paper?

If you can answer no to all of the above, write 'I declare that I have no competing interests' below. If your reply is yes to any, please give details below.

declare that I have no competing interests

I agree to the open peer review policy of the journal. I understand that my name will be included on my report to the authors and, if the manuscript is accepted for publication, my named report including any attachments I upload will be posted on the website along with the authors' responses. I agree for my report to be made available under an Open Access Creative Commons CC-BY license (http://creativecommons.org/licenses/by/4.0/). I understand that any comments which I do not wish to be included in my named report can be included as confidential comments to the editors, which will not be published.

I agree to the open peer review policy of the journal

To further support our reviewers, we have joined with Publons, where you can gain additional credit to further highlight your hard work (see: https://publons.com/journal/530/gigascience). On publication of this paper, your review will be automatically added to Publons, you can then choose whether or not to claim your Publons credit. I understand this statement.

Yes
